# IL-1β/CXCL12 signalling orchestrates adipocyte–pancreatic neuroendocrine tumor crosstalk

**DOI:** 10.1186/s12967-026-08428-z

**Published:** 2026-06-18

**Authors:** Monica Oldani, Elisa Stellaria Grassi, Germano Gaudenzi, Ilona Rybinska, Silvia Carra, Elena Massardi, Nicola Fazio, Michele Caraglia, Luca Persani, Giovanni Vitale

**Affiliations:** 1 Laboratory of Geriatric and Oncologic Neuroendocrinology Research, IRCCS, Istituto Auxologico Italiano, 20100 Milan, Italy; 2 Laboratory of Endocrine and Metabolic Research, IRCCS, Istituto Auxologico Italiano, 20100 Milan, Italy; 3 Department of Medical Biotechnology and Translational Medicine, University of Milan, 20122 Milan, Italy; 4 Division of Gastrointestinal Medical Oncology and Neuroendocrine Tumors, European Institute of Oncology, IEO, IRCCS, ENETS and EURACAN NEN Center of Excellence, Milan, Italy; 5 Department of Precision Medicine, University of Campania “Luigi Vanvitelli”, Via L. De Crecchio 7, 80138 Naples, Italy; 6 Laboratory of Precision and Molecular Oncology, Biogem Scarl, Institute of Genetic Research, Contrada Camporeale, 83031 Ariano Irpino, Italy

**Keywords:** Pancreatic neuroendocrine tumors, Adipocyte–tumor crosstalk, Cancer-associated adipocytes, IL-1β, CXCL12/CXCR4 axis

## Abstract

**Background:**

Pancreatic neuroendocrine tumors (PanNETs) are rare neoplasms belonging to the broader group of neuroendocrine neoplasms. Epidemiological evidence indicates that visceral obesity is associated with both an increased incidence of PanNETs and unfavorable clinicopathological features. However, the mechanisms underlying adipocyte–PanNETs interactions remain poorly understood.

**Methods:**

Here, we investigated the crosstalk between adipocytes and PanNET cells (BON-1 and QGP-1 cell lines) using indirect co-culture systems and conditioned media derived from differentiated 3T3–L1 adipocytes or PanNET cells.

**Results:**

Our results demonstrate that PanNET cells induce adipocyte reprogramming toward a cancer-associated adipocyte (CAA) phenotype, characterized by lipid depletion and downregulation of adipogenic markers, including *Pparg, Fabp4, Hsl*, and *Atgl*. We identified PanNET-derived interleukin-1β (IL-1β) as a key driver of adipocyte conversion into CAAs. This process was accompanied by increased secretion of the chemokine CXCL12 from CAAs, which in turn appeared to enhance PanNET cell proliferation and IL-1β release, thereby establishing a positive bidirectional crosstalk consistent with a feedback-like mechanism between CXCL12 and IL-1β in CAAs and PanNET cells, respectively. Indeed, pharmacological disruption of this axis using AMD3100, a CXCR4 antagonist, significantly reduced both IL-1β and CXCL12 secretion, prevented adipocyte reprogramming, and suppressed tumor cell proliferation. Comparable effects were observed following incubation of PanNET–adipocyte co-cultures with canakinumab, an IL-1β pathway inhibitor.

**Conclusion:**

Collectively, these findings identify IL-1β and CXCL12 as potential critical mediators of the inflammatory crosstalk between adipocytes and PanNET cells. Targeting this signalling axis may therefore represent a promising therapeutic strategy for PanNETs.

**Graphical Abstract:**

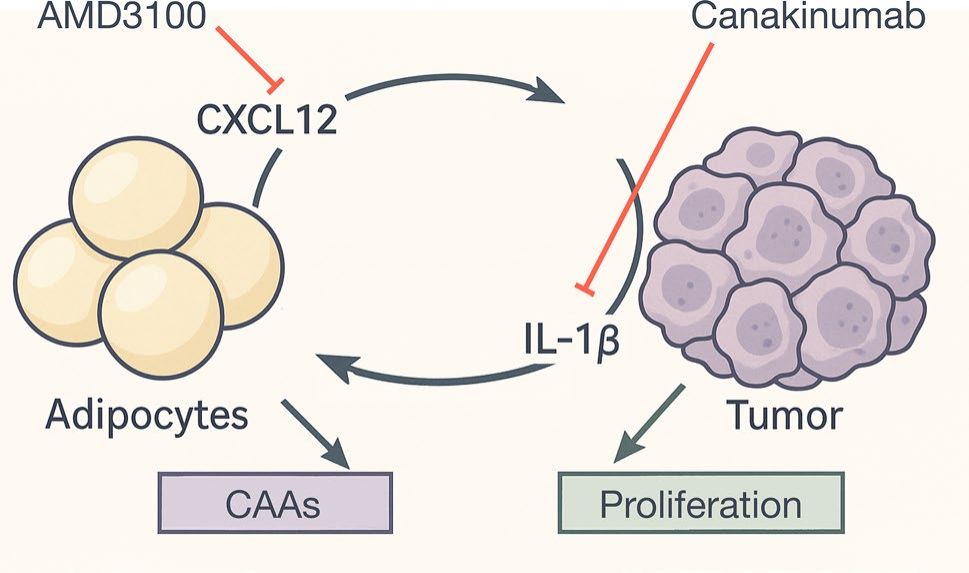

**Supplementary Information:**

The online version contains supplementary material available at 10.1186/s12967-026-08428-z.

## Introduction

Pancreatic neuroendocrine tumors (PanNETs) are rare neoplasms, accounting for less than 3% of all primary pancreatic malignancies [1–3]. Although PanNETs are often considered indolent, their biological and clinical behavior is highly heterogeneous. A subset of PanNETs displays a more aggressive phenotype, with a propensity for disease progression, limited response to conventional therapies, and reduced survival outcomes [4, 5]. Despite their generally slow growth rate, PanNETs metastasize in approximately 50–80% of cases [6]. These metastatic patterns contribute to a median progression-free survival of less than 11 months in advanced-stage disease [7]. Currently available therapeutic options, including somatostatin analogues, peptide receptor radionuclide therapy, mTOR inhibitors, anti-angiogenic agents targeting VEGF-A and its receptor, tyrosine kinase inhibitors, and chemotherapy, have shown modest and often transient clinical benefit [4, 8]. These limitations underscore the need to identify novel therapeutic targets and strategies to improve patient outcomes.

Epidemiological studies have reported that visceral adiposity is associated with shorter progression-free survival in well-differentiated gastro-entero-pancreatic neuroendocrine neoplasms, while obese or diabetic patients with PanNETs more frequently present with advanced or metastatic disease [9–11]. At the same time, fat accumulation within pancreatic tissue, a condition known as *fatty pancreas*, has been associated with an increased risk of pancreatic ductal adenocarcinoma (PDAC) and with poorer prognosis [12, 13]. In this context, adipocytes contribute to tumor progression by releasing adipokines and pro-inflammatory mediators that remodel the tumor microenvironment, as observed in other tumor types [14–17].

Given the anatomical proximity of visceral fat depots to the pancreas, adipose tissue may similarly influence PanNET biology. Although direct evidence in PanNETs remains limited, a bidirectional crosstalk between adipocytes and neuroendocrine tumor cells is likely to contribute to tumor progression. Notably, such interactions have already been described in prostate, ovarian, and colon cancers, where tumor cells reprogram neighboring adipocytes into cancer-associated adipocytes (CAAs) [15–17]. These reprogrammed adipocytes secrete pro-tumorigenic cytokines, remodel extracellular matrix components, and promote tumor cell migration, invasion, and resistance to therapy [18, 19].

In light of these considerations, we investigated the interplay between adipocytes and PanNET cells using the human PanNET cell lines BON-1 and QGP-1. To this end, we employed both indirect co-culture systems and conditioned media derived from differentiated 3T3–L1 adipocytes or from BON-1 and QGP-1 tumor cells, respectively.

## Material and methods

### Cell lines

BON-1 and QGP-1 cell lines were kindly provided by Dr Valeria Giandomenico, Department of Medical Sciences, Endocrine Tumor Biology, Uppsala University, Uppsala, Sweden. BON-1 and QGP-1, two widely used human PanNET cell lines, were derived from a pancreatic carcinoid tumor lymph node metastasis and a primary pancreatic islet cell carcinoma, respectively [20, 21]. The 3T3–L1 murine preadipocyte cell line (ATCC® CCL-153™, Manassas, VA, USA), derived from mouse embryonic fibroblasts, was obtained from the American Type Culture Collection. BON-1 and QGP-1 cells were cultured in GlutaMAX^TM^ DMEM supplemented with 10% FBS, 2 mM L-glutamine, and 2 mM penicillin/streptomycin (P/S), following the manufacturer’s recommendations. 3T3–L1 cells were maintained in DMEM/F12 medium with 2 mM L-glutamine and 2 mM P/S. To slow fibroblast proliferation, 3T3–L1 cultures were maintained in 2% FBS. 3T3–L1 cells were not used beyond passage 8, due to reduced differentiation efficiency at higher passages. All cells were cultured at 37 °C in a humidified incubator with 5% CO₂. Cells were subcultured before reaching confluence; at approximately 80% confluence, they were detached using trypsin and reseeded according to experimental needs. All cell lines were used at low passages, routinely tested for mycoplasma contamination using standard detection methods, and confirmed to be mycoplasma-free prior to use in experiments.

### Adipocyte differentiation

3T3–L1 cells are a well-established model for studying adipocyte biology due to their highly standardized differentiation protocols. In our experiments, 3 × 10^5^, 8 × 10^4,^ or 6 × 10^4^ cells per well were seeded in 6-, 12-, or 24-well plates, respectively, and allowed to reach confluence over the course of three days. On day 0, differentiation was induced by replacing the culture medium with the Adipogenesis Differentiation Medium (AMSBIO, AMS Biotechnology, Cambridge, MA). To enhance differentiation efficiency, the medium was further supplemented with 2 µg/mL rosiglitazone (Cayman Chemical, Ann Arbor, MI, USA), a PPARγ agonist [22]. The same medium change was performed again on day 1. On day 3, the medium was replaced with standard 3T3–L1 growth medium (10% FBS) supplemented with 1 µg/mL insulin (Sigma-Aldrich, MO, USA). After 48 hours, insulin was withdrawn, and cells were cultured in high-glucose DMEM containing 10% FBS, 2 mM L-glutamine, and 2 mM penicillin/streptomycin (P/S) (all purchased from Sigma). The medium was changed every three days thereafter. Mature adipocytes were obtained after 14 days of differentiation.

### Preparation of conditioned medium

*Fibroblast-conditioned medium.* A total of 3 × 10^5^ cells per well were seeded in 6-well plates. On day 3, when cells had reached confluence, fibroblasts were gently washed with PBS, and an appropriate volume of standard growth medium was added to each well. After 24 hours of incubation, the medium conditioned by 3T3-L1 fibroblasts (M-3T3-L1) was collected.

*Adipocyte-conditioned medium*. On day 13 of the differentiation protocol, adipocytes were gently washed with PBS, and an appropriate volume of high-glucose DMEM was added to each well of a 6-well culture plate. After 24 hours of incubation, the medium conditioned by 3T3-L1 mature adipocytes (M-AD) was collected.

*PanNET cell line-conditioned medium*. A total of 1.5 × 10^6^ BON-1 or QGP-1 cells were seeded in 6-well culture plates. After three days, the medium was replaced with a fresh complete growth medium. The conditioned media, referred to as M-BON-1 and M-QGP-1, respectively, were collected after an additional 24 hours.

In all cases, the collected conditioned media were filtered to remove cellular debris and stored at −80 °C until use.

### Treatments with conditioned-media

*Fibroblast and adipocytes experiments:* Confluent 3T3-L1 fibroblasts, (three days post-seeding) and adipocytes (at the end of the differentiation protocol), were incubated with a 1:1 mixture of either M-BON-1 or M-QGP-1 conditioned medium and standard growth medium for 72 hours. Cells treated under these conditions were subsequently used for downstream analyses.

*PanNET cell lines experiments: MTT assay.* The effects of M-3T3-L1 and M-AD on BON-1 and QGP-1 cells were evaluated to determine the working concentration. BON-1 and QGP-1 cells were seeded at a density of 6 × 10^3^ cells per well in 96-well culture plates. After 24 hours, tumor cells were treated with increasing concentrations of M-3T3-L1 or M-AD (5, 25, 50, 75, and 100%) diluted in standard tumor growth medium. Cells cultured in standard growth medium alone served as controls. After 72 hours of incubation, the medium was removed and replaced with 110 µL of MTT working solution (composed of 10 µL of 5 mg/mL MTT in PBS and 100 µL of culture medium) per well. After 4 hours of incubation at 37 °C, the MTT solution was discarded, and 100 µL of 0.1 N hydrochloric acid (HCl) in isopropanol was added to each well to solubilize the formazan crystals. Absorbance was measured at 570 nm using a microplate reader.

*Proliferation assay.* BON-1 and QGP-1 cells were seeded in 96-well plates at a density of 2 × 10^4^ cells per well, in duplicate for each time point and experimental condition. On day two, cells were treated with a 1:1 mixture of M-3T3-L1 or M-AD and standard maintenance medium. Control wells received standard growth medium alone. Cells were harvested at 24, 48, and 72 hours post-treatment. For detachment, 40 µL of trypsin was added to each well and neutralized with 140 µL (24 h), 160 µL (48 h), or 180 µL (72 h) of complete medium. The detached cells were collected and counted manually under a microscope to assess the rate of cell proliferation.

### Co-culture experiments

Depending on the type of culture plate used (6-, 12-, or 24-well), fibroblasts or adipocytes were prepared according to the protocols described in Sections. “Preparation of conditioned medium” and “Adipocyte differentiation”, respectively. 3T3–L1 cells were always seeded at the bottom of multiwell culture plates. BON-1 and QGP-1 cells were seeded in the upper compartment of a ThinCerts^TM^, pore size 0.4 µm (Greiner Bio-One, Frickenhausen, Germany) at a density of 1.5 × 10^6^, 7.5 × 10^5^, or 8 × 10^4^ cells per insert for 6-, 12-, and 24-well culture plates, respectively. BON-1 and QGP-1 cells were co-cultured in 2% FBS DMEM/F12 complete with fibroblasts or in complete high-glucose DMEM medium in the presence of adipocytes for 72 hours.

*Proliferation assay.* This experiment was performed using 24-well plates and in duplicate for each time point and experimental condition. Control cells received standard growth medium alone. Cells were harvested at 24, 48, and 72 hours post-incubation. For detachment, 40 µL of trypsin was added to each insert and subsequently neutralized with 140 µL (24 h), 160 µL (48 h), or 180 µL (72 h) of complete medium. The detached cells were collected and manually counted under a microscope to evaluate cell proliferation.

### Oil Red O staining

Oil Red O staining was performed to assess intracellular lipid accumulation in differentiated 3T3–L1 adipocytes. At the end of the experiments, cells were gently washed twice with PBS and fixed with 4% paraformaldehyde (PFA) for 30 minutes at room temperature (RT). Following fixation, cells were washed again with PBS and incubated with 60% isopropanol for 15 minutes to prepare the cells for staining. The isopropanol was then removed, and the cells were allowed to air dry before staining. Oil Red O working solution (prepared by diluting a stock solution of 0.5% Oil Red O in isopropanol with distilled water at a 6:4 ratio, then filtered before use) was added to each well and incubated for 2 hours at room temperature. After staining, cells were washed thoroughly with distilled water until the background was clear. Stained lipid droplets were visualized under a light microscope. For quantification, the retained dye was eluted using 100% isopropanol, and absorbance was measured at 540 nm using a microplate reader.

### Lipolysis assessment

Lipolytic activity was assessed using the Lipolysis (3T3–L1) Colorimetric Assay Kit (Sigma-Aldrich, MO, USA), following the manufacturer’s instructions. After 14 days of differentiation, 3T3–L1 adipocytes cultured in 24-well plates were washed with 300 µL of Lipolysis Wash Buffer. Subsequently, 350 µL of Lipolysis Assay Buffer was added to each well, and cells were stimulated with 10 µM isoproterenol, a β-adrenergic receptor agonist, for 2 hours at 37 °C to induce lipolysis. After stimulation, 50 µL of the supernatant from each well was transferred to a 96-well plate. Reaction mixes were prepared as per the kit protocol and added to the corresponding wells. 50 µL of these mixes was added to the sample wells and incubated at RT for 30 minutes, protected from light. Standards were prepared using serial dilutions of the provided 100 mM glycerol standard, and blanks without the enzyme mix were included for background correction. Absorbance was measured at 570 nm using a microplate reader.

### RT-qPCR

Total RNA was extracted from cells cultured in 6-well plates using 300 µL of TRIzol™ Reagent (Invitrogen™, MA, USA) per well. An equal volume of pure ethanol was added, and the mixture was processed using the Quick-RNA Miniprep Kit (Zymo Research, Irvine, CA), following the manufacturer’s instructions. RNA was eluted in 50 µL of RNase-free water. cDNA synthesis was performed using the GoScript™ Reverse Transcriptase System (Promega, USA), with 500 ng of total RNA per sample. Quantitative RT-PCR (RT-qPCR) was conducted using a QuantStudio Real-Time PCR System (Thermo Fisher Scientific, MA, USA). RT-qPCR was performed using PowerUp SYBR Green Master Mix according to the manufacturer’s instructions: in a final reaction volume of 20 µL, containing 10 µL of 2X master mix, gene-specific primers, and cDNA template. Primers were used at a final concentration of 250 nM each. The amplification protocol consisted of an initial incubation at 50 °C for 2 min and 95 °C for 2 min, followed by 40 cycles of 95 °C for 15 s and 60 °C for 1 min. Relative gene expression was calculated using the 2^−ΔΔCt method. Primer sequences are listed in Table 1.

**Table 1:** Primer sequences used for RT-qPCR

Gene Symbol	Gene Name	Forward	Reverse
*Lep*	Leptin	5’-GCAGTGCCTATCCAGAAAGTCC-3’	5’-GGAATGAAGTCCAAGCCAGTGAC-3’
*Adipoq*	Adiponectin	5’-TGGAATGACAGGAGCTGAAGG-3’	5’-TATAAGCGGCTTCTCCAGGCT-3’
*Pparg*	Peroxisome Proliferator-Activated Receptor Gamma	5’-CCGAAGAACCATCCGATTGA-3’	5’-TTTGTGGATCCGGCAGTTAAG-3’
*Fabp4*	Fatty Acid Binding Protein 4	5’-AAACTGGTGGTGGAATGCGT-3’	5’-GCGAACTTCAGTCCAGGTCA-3’
*Fads1*	Fatty acid desaturase 1	5’-CGCCAAACGCGCTACTTTACTTG-3’	5’-CATCCTGACCCGCGTAGTGG-3’
*Hsl (Lipe)*	Hormone-sensitive lipase	5’-GGCTTACTGGGCACAGATACCT-3’	5’-CTGAAGGCTCTGAGTTGCTCAA-3’
*Atgl (Pnpla2)*	Adipose triglyceride lipase	5’-CTGAGAATCACCATTCCCACATC-3’	5’-CACAGCATGTAAGGGGGAGA-3’
*IL-6*	Interleukin 6	5’-TACCACTTCACAAGTCGGAGGC −3’	5’-CTGCAAGTGCATCATCGTTGTTC −3’
*α-SMA (Acta2)*	Actin Alpha 2, Smooth Muscle	5’-GGAGAAGCCCAGCCAGTCGC −3’	5’-AGCCGGCCTTACAGAGCCCA-3’
*Gapdh*	Glyceraldehyde-3-phosphate dehydrogenase	5’-GGGTCCCAGCTTAGGTTCAT-3’	5’- TACGGCCAAATCCGTTCACA-3’

All genes included in Table 1 were analyzed as genes of interest (GOI), except for GAPDH, which served as the reference (housekeeping) gene for normalization

### Immunofluorescence and confocal microscopy analysis

3T3–L1 cells, seeded onto fibronectin (diluted 1:25 in PBS)-coated glass coverslips (Sigma-Aldrich, MO, USA), were differentiated into adipocytes as described in Section “Adipocyte differentiation”. Coverslips were incubated for 30 minutes at 37 °C and excess solution was removed prior to cell seeding. Co-culture was established following the protocol outlined in Section “Co-culture experiments”, using a 12-well culture plate. On the day of the experiment, coverslips were washed twice with warm PBS and then fixed with 4% PFA/PBS for 10 minutes at RT. After two washes with PBS, cells were permeabilized with 0.1% Triton-X100/PBS solution for 10 minutes RT. After permeabilization, the slides were washed with PBS twice, blocked in 5% BSA/PBS solution for 1 hour RT and then incubated with anti-α-SMA (A2547-2 ML, Sigma Aldrich, 1:150 in 5% BSA/PBS) overnight at 4 °C. The following day coverslips were washed twice with PBS and then incubated with an anti-mouse Alexa-fluor 488 conjugated secondary antibody (Thermo scientific, 1:250 in 5% BSA/PBS) for 1 h at RT. After one wash with PBS, slides were incubated with 594 Alexa-Fluor conjugated phalloidin (Thermo scientific, 1x solution in 1% BSA) for 20 mins followed by incubation with 2 µg/mLl DAPI/PBS for 20 minutes RT. Slides were then washed once in PBS and mounted with FluorSave Reagent (Merck Millipore). Images were acquired with Nikon EclipseTi-E inverted microscope equipped with IMA10X Argon-ion laser system by Melles Griot (Nikon). A Z-stack of the whole thickness was acquired through NIS Element Imaging Software version 5.02.

For cell types quantifications, for each 20x Z-stack, cells were attributed to one of two populations, adipo-like or fibro-like and cytoplasmic integrated densities of α-SMA and F-actin measured with Cell Profiler [23, 24]. Briefly, nuclei were defined as primary objects in the DAPI channel as nuclear dimensions 25–60 (fibro-like) or 16–24 (adipo-like), based on the previously determined median dimensions of cell types nuclei. Secondary objects were determined by propagation (FIBRO-like) or by watershed-gradient (ADIPO-like) methods with global threshold strategy in the F-actin channel. Cytoplasm was defined as tertiary objects resulting from subtraction of nuclear area from the respective secondary object. α-SMA and F-Actin colocalization was determined on single plane 100x scansions.

### Zebrafish PanNET transplantable model

Adult zebrafish of the *Tg(fli1a:EGFP)*^*y1*^ line were maintained in accordance with both European directives (2010/63/EU) and Italian laws (Legislative Decree 26/2014) governing the use of live animals in research. Xenograft experiments were performed on zebrafish embryos and larvae up to 5 days post-fertilization (dpf). Embryos were obtained via natural spawning and subsequently staged and cultured at 28 °C in fish water (containing 0.1 g/L NaHCO3, 0.1 g/L Instant Ocean, and 0.192 g/L CaSO4•2H2O), supplemented with 0.003% PTU (1-phenyl-2-thiourea; Sigma-Aldrich, Saint Louis, MO) to inhibit pigmentation and 0.01% methylene blue to prevent fungal contamination.

BON-1 and QGP-1 cells, grown in monoculture and co-culture with adipocytes, were labeled with the red fluorescent vital dye CM-DiI (Invitrogen, Carlsbad, CA, USA) and subsequently resuspended in PBS. At 2 dpf, approximately 500 tumor cells were xenografted into zebrafish *Tg(fli1a:EGFP)*^*y1*^ embryos in the subperidermal cavity, located between the periderm and the yolk syncytial layer, in close proximity to the sub-intestinal vessel (SIV) plexus. Cell implantation was performed using a FemtoJet microinjector (Eppendorf, Hamburg, Germany) coupled to an InjectMan NI 2 micromanipulator (Eppendorf, Hamburg, Germany). Accurate deposition of the cell suspension at the designated site was critical to induce a consistent angiogenic response. Embryos in which cells were implanted into the yolk were excluded in subsequent analyses. As a control of the xenotransplantation procedure we considered embryos injected only with PBS, the cellular resuspension solution. Following transplantation, embryos were maintained at 32 °C, a temperature representing a compromise between optimal zebrafish development (28 °C) and conditions favorable for mammalian cell viability and metabolic activity (37 °C). At 1 day post-injection (dpi), implanted embryos were imaged using an epifluorescence microscope (Leica M205FA; Wetzlar, Germany) equipped with a digital camera (Leica DFC450C; Wetzlar, Germany). Identical acquisition parameters were applied to all samples. Using ImageJ2 software, a region of interest (ROI) was defined for each embryo in the area surrounding the xenograft, selectively encompassing endothelial structures sprouting from the SIV plexus and/or the common cardinal vein (CCV) while excluding vessels formed as part of normal development. The same threshold was applied to the EGFP channel across all embryos, minimizing background noise and restricting the area calculation within the defined threshold for each ROI. The area of the EGFP signal was considered as an arbitrary unit of tumor-induced angiogenesis. Data were normalized against the mean observed in BON-1 and QGP-1 grown alone, which was set to 1.0. All experiments were conducted three times, with a minimum of 15–20 embryos in each experimental group.

### Cytokine quantification by ELISA

CXCL12 and Human IL-1β secretion levels were measured in the cell culture supernatants using a mouse SDF-1/CXCL12 (Stromal Cell Derived Factor 1) ELISA kit (Elabscience, Houston, Texas), and the Human IL-1 beta Sandwich ELISA kit (Proteintech, IL, USA), following the manufacturer’s instructions.

### Pharmacological inhibition

To disrupt CXCL12-mediated signaling, AMD3100 (Plerixafor, MedChem, NJ, USA), a selective C-X-C chemokine receptor type 4 (CXCR4) antagonist, was used. In parallel, to confirm that the observed transformation was mediated by IL-1β signaling, canakinumab (Ilaris, MedChem, NJ, USA), a monoclonal antibody with the specific ability to bind and inhibit the action of IL-1β, was employed. In a MTT assay, BON-1 and QGP-1 cells were seeded at a density of 6 × 10^3^ cells per well in 96-well plates. After 24 hours, cells were treated with AMD3100 at concentrations of 10, 25, 50, and 100 ng/mL, and with canakinumab at 1, 10, 100, and 1000 ng/mL. Vehicle-treated cells (0.5% ethanol for AMD3100; PBS for canakinumab) served as controls. Treatments were carried out for 72 hours. Among the tested doses, 10 and 25 ng/mL of AMD3100 were well tolerated and selected for subsequent experiments. No significant toxicity was observed at any of the canakinumab concentrations tested; therefore, 100 ng/mL was chosen for further assays.

### Statistical analyses

Statistical evaluations were carried out using GraphPad Prism (version 8.5; GraphPad Software, La Jolla, CA, USA). A threshold of *p* < 0.05 was used to define statistical significance. Normality of data distribution was assessed using the Shapiro–Wilk test. When data followed a normal distribution, differences between two groups were analyzed using the Student’s *t*-test, while comparisons among three or more groups were evaluated using either one-way or two-way ANOVA, depending on the experimental design. Dunnett’s, Tukey’s, or Sidak’s post hoc test was used to assess pairwise differences when ANOVA indicated statistical significance. For non-normally distributed data, differences between two groups were analyzed using the Mann–Whitney U test. The Kruskal–Wallis test was applied for group comparisons, followed by Dunn’s multiple comparison test to identify significant differences between groups. All experiments were independently repeated at least three times, and results are presented as mean ± SEM in the figures.

## Results

### Tumor-secreted factors adjust adipocyte morphology and gene transcription

**Fig. 1:**
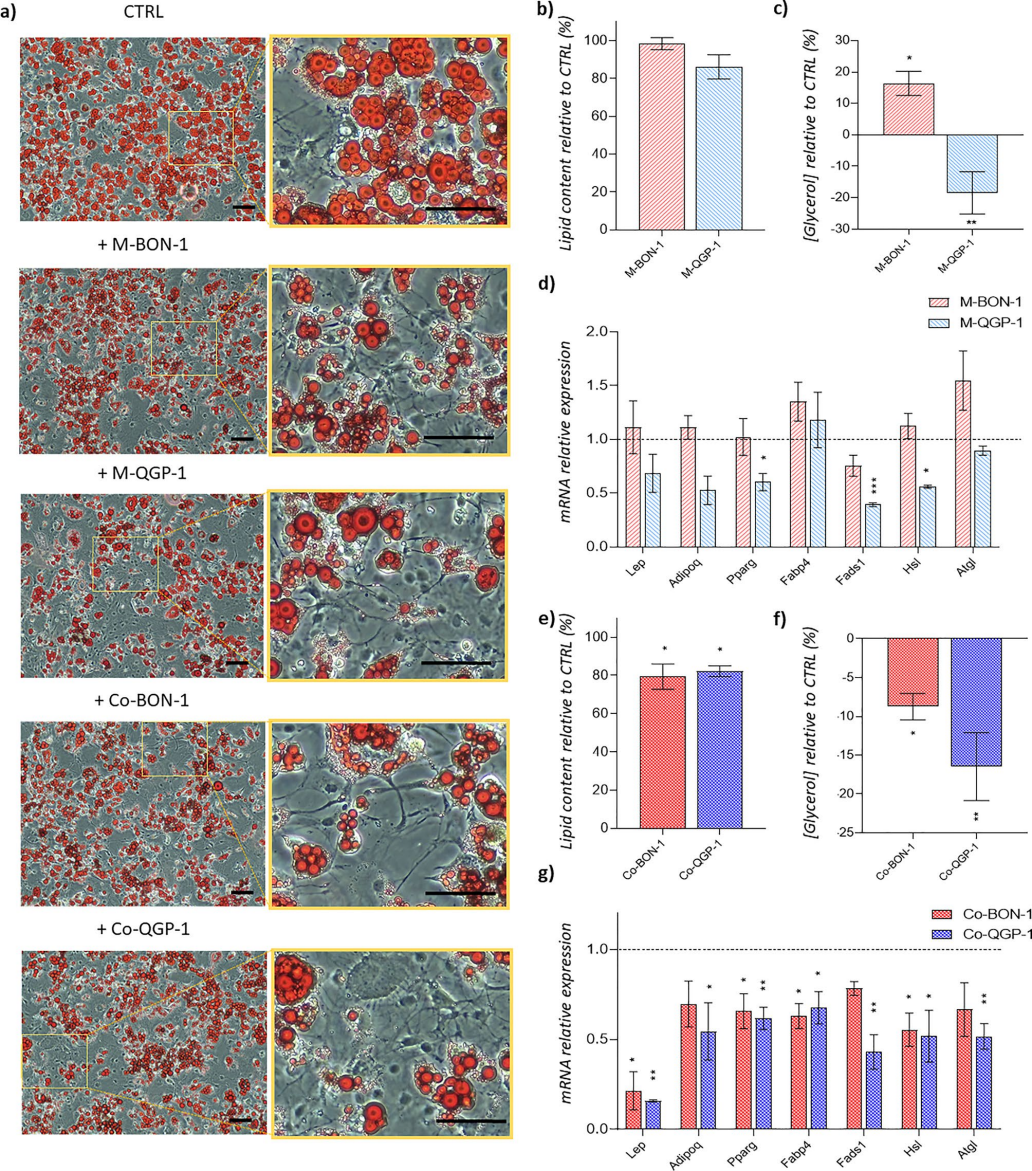
PanNET tumor cells alter adipocyte morphology, lipid content, lipolytic activity, and gene expression. (**a**) Representative brightfield images (10× magnification) of differentiated 3T3–L1 adipocytes cultured alone (CTRL), incubated with conditioned-media from BON-1 and QGP-1 (M-BON-1 and M-QGP-1), or indirectly co-cultured for 72 hours with PanNET tumor cell lines BON-1 and QGP-1 (co-BON-1 and Co-QGP-1). Prior to imaging, adipocytes were fixed and stained with Oil Red O to visualize intracellular lipid droplets. A magnified view (yellow box) highlights the presence of fibroblast-like cells. Scale bars: 100 μm (main images and magnifications); (**b**) Quantification of intracellular lipid accumulation based on spectrophotometric measurement of Oil Red O extracted from stained adipocytes. Results are expressed as relative lipid content (%) normalized to CTRL; (**c**) Induced lipolysis was assessed by stimulating adipocytes with 10 μM isoproterenol for 2 hours. The concentration of glycerol released into the culture medium was measured as an indicator of lipolytic activity and is presented relative to CTRL; (**d**) Gene expression analysis of adipocyte identity and lipid metabolism markers was performed by RT-qPCR. Expression levels of *Lep*, *Adipoq*, *Pparg*, *Fabp4*, *Fads1*, *Hsl*, and *Atgl* were quantified and normalized to housekeeping gene. Fold changes were calculated relative to adipocytes cultured alone (CTRL); (**e**) Quantification of Oil Red O staining in adipocytes grown under co-culture conditions (co-BON-1 and Co-QGP-1). Results are expressed as relative lipid content (%) normalized to CTRL; (**f**) Quantification of isoproterenol-induced glycerol release into the culture medium under both co-culture conditions, compared with adipocytes cultured alone; (**g**) Expression analysis of *Lep*, *Adipoq*, *Pparg*, *Fabp4*, *Fads1*, *Hsl*, and *Atgl* in adipocytes grown under co-culture conditions by RT-qPCR. Fold changes were calculated relative to adipocytes cultured alone (CTRL). Statistical analysis was performed using Kruskal-Wallis test followed by Dunn’s multiple comparisons test in Oil Red O assays (**b** and **e**), one-way ANOVA followed by Tukey’s multiple comparisons test for the glycerol quantification (**c** and **f**), paired t-test for all genes in RT-qPCR assays (**d** and **g**). In all sections, data represent mean ± SEM of at least three independent experiments. * *p* < 0.05, ** *p* < 0.01, *** *p* < 0.001 were considered statistically significant

We first assessed the effects of tumor-conditioned medium on adipocytes to determine whether tumor-secreted factors alone were sufficient to trigger adipocyte dedifferentiation into CAAs. As shown in Fig. 1a, both M-BON-1 and M-QGP-1 induced morphological alterations in adipocytes, leading to the appearance of a subpopulation of cells exhibiting a fibroblast-like phenotype, as highlighted in the enlarged panel (Fig. 1a, yellow inset). However, adipocytes exposed to M-BON-1 showed no significant changes in the expression of adipocyte-specific and lipid metabolism–related genes, retaining their lipid content, and displaying a significant increase in glycerol release compared to untreated controls (Fig. 1b, c, and d). In contrast, the morphological transition, particularly evident in adipocytes exposed to M-QGP-1, was associated with a tendency toward reduction of intracellular lipid stores and a significantly blunted lipolytic response to β-adrenergic stimulation (Fig. 1b and c). These findings are mechanistically linked, as the decreased glycerol release upon isoproterenol treatment likely reflects substrate depletion caused by prior triglyceride loss during the M-QGP-1 incubation. Moreover, a significant downregulation of Peroxisome Proliferator-Activated Receptor Gamma (*Pparg)*, a master regulator of adipocyte identity, as well as of genes involved in lipid metabolism and lipolysis, such as Fatty acid desaturase (*Fads1)* and Hormone-sensitive lipase (*Hsl),* provided further evidence that M-QGP-1-derived molecules were able to induce adipocyte reprogramming toward a CAA-like phenotype (Fig. 1d).

To additionally investigate the effects of PanNET cell lines on adipocytes, we employed an indirect co-culture system that allowed continuous bidirectional communication between the two cell types via secreted factors. Under co-culture conditions (Co-BON-1 and Co-QGP-1), in addition to the presence of branched cells with a fibroblast-like morphology, optical microscopy revealed a marked reduction in lipid droplet size (Fig. 1a, yellow enlarged section), quantitatively supported by a significant decrease in Oil Red O staining (Fig. 1e). The reduction in intracellular lipid content was accompanied by a significant decrease in isoproterenol-induced glycerol release into the culture medium under both co-culture conditions, compared with adipocytes cultured alone (Fig. 1f). Moreover, at the molecular level, adipocytes subjected to co-culture began to lose their characteristic gene expression profile. Specifically, interaction with both tumor cell lines resulted in a significant downregulation of Leptin (*Lep), Pparg,* Fatty Acid Binding Protein 4 (*Fabp4)* and *Hsl*. In addition, reduced expression of Adiponectin *(Adipoq), Fads1*, and Adipose triglyceride lipase *(Atgl)* further indicated a profound disruption of adipocytes lipid metabolism, particularly in adipocytes co-cultured with QGP-1 cells (Fig. 1g). Taken together, the reduction in intracellular lipid content and the loss of classical adipocyte markers strongly indicate that adipocytes were undergoing a phenotypic shift consistent with CAAs. These changes were more pronounced under co-culture conditions than with treatment using conditioned media alone, suggesting that continuous tumor–adipocyte crosstalk drives a stronger reprogramming effect.

### Emergence of fibroblast-like cells with pro-inflammatory features from adipocytes in PanNET Co-culture systems

**Fig. 2:**
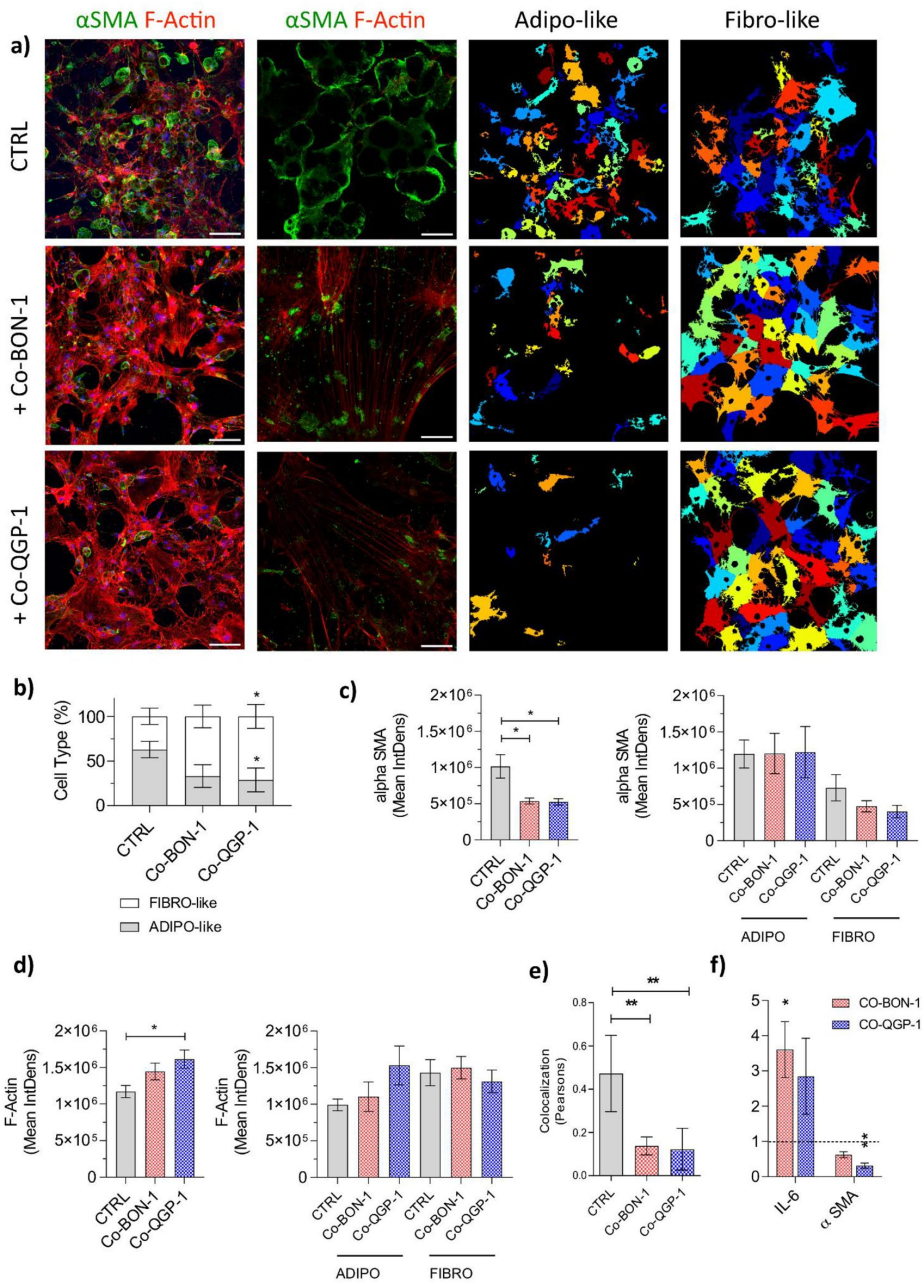
Phenotypic and molecular characterization of adipocytes exposed to tumor cells in co-culture. (**a**) Representative confocal microscopy images of whole Z-stack (20x magnification; scale Bar: 100 μm) and F-actin fibers single plane detail (100x magnification; scale Bar: 20 μm) of differentiated 3T3–L1 adipocytes cultured alone (CTRL), or indirectly co-cultured for 72 hours with BON-1 or QGP-1 tumor cells (co-BON-1 and Co-QGP-1). F-actin is shown in red, and α-SMA in green. Nuclei were counterstained with DAPI (blue). (**b**) Quantification of the percentage of cells classified as “adipocyte-like” (ADIPO-like) or “fibroblast-like” (FIBRO-like) by image analysis software. **c**–**d**) quantification of mean integrated density for α-SMA (**c**) and F-actin (**d**), both across the entire image and separately within the distinct cell populations identified. (**e**) Co-localization analysis between α-SMA and F-actin single plane signals. (**f**) Relative mRNA expression levels of IL-6 and α-SMA assessed by RT-qPCR, normalized to housekeeping genes. Fold changes were calculated relative to untreated adipocytes (CTRL). Statistical analysis was performed using one-way ANOVA followed by appropriate post hoc tests for panels b–e, and paired t-test for gene expression analysis in f. All data are presented as mean ± SEM from at least three independent experiments. * *p* < 0.05, ** *p* < 0.01, *** *p* < 0.001 were considered statistically significant

Based on previous observations, we morphologically evaluated adipocytes cultured under standard conditions or indirectly co-cultured with tumor cells using confocal microscopy, as shown in Fig. 2. After three days, we observed a marked increase in the proportion of fibroblast-like cells, accompanied by a concomitant decrease in adipocyte-like cells, suggesting that tumor-derived signals promoted stromal remodeling through adipocyte dedifferentiation (Fig. 2a and b). To additionally characterize these fibroblast-like cells, we evaluated the expression of canonical cancer-associated fibroblast (CAF) markers to identify the phenotypic transition occurring in our CAAs. As illustrated in Fig. 2a and c, co-cultured adipocytes exhibited a progressive decrease in α-SMA expression compared to unexposed adipocytes (CTRL), and this change affected mostly cells with fibro-like morphology. Additionally, we observed a pronounced reorganization of the actin cytoskeleton, characterized by the appearance of stress fibers, which additionally supports the transition toward a fibroblast-like reactive phenotype (Fig. 2a and d). Notably, α-SMA did not colocalize with F-actin fibers, suggesting the absence of the organized contractile structures typically found in myofibroblast-like CAFs (Fig. 2e). On the other hand, these cells exhibited features more consistent with inflammatory CAFs (iCAFs), which are defined by low α-SMA expression, and higher expression of IL-6 (Fig. 2f) [25]. However, due to the functional and molecular similarities between CAAs and CAFs, and considering that our co-culture experiments spanned only three days, it is plausible that the observed transformation is still in progress rather than fully achieved. Accordingly, we will refer to these cells as CAF-like cells with pro-inflammatory features.

### Adipocyte co-cultured more potently stimulates PanNET proliferation compared to conditioned medium

**Fig. 3:**
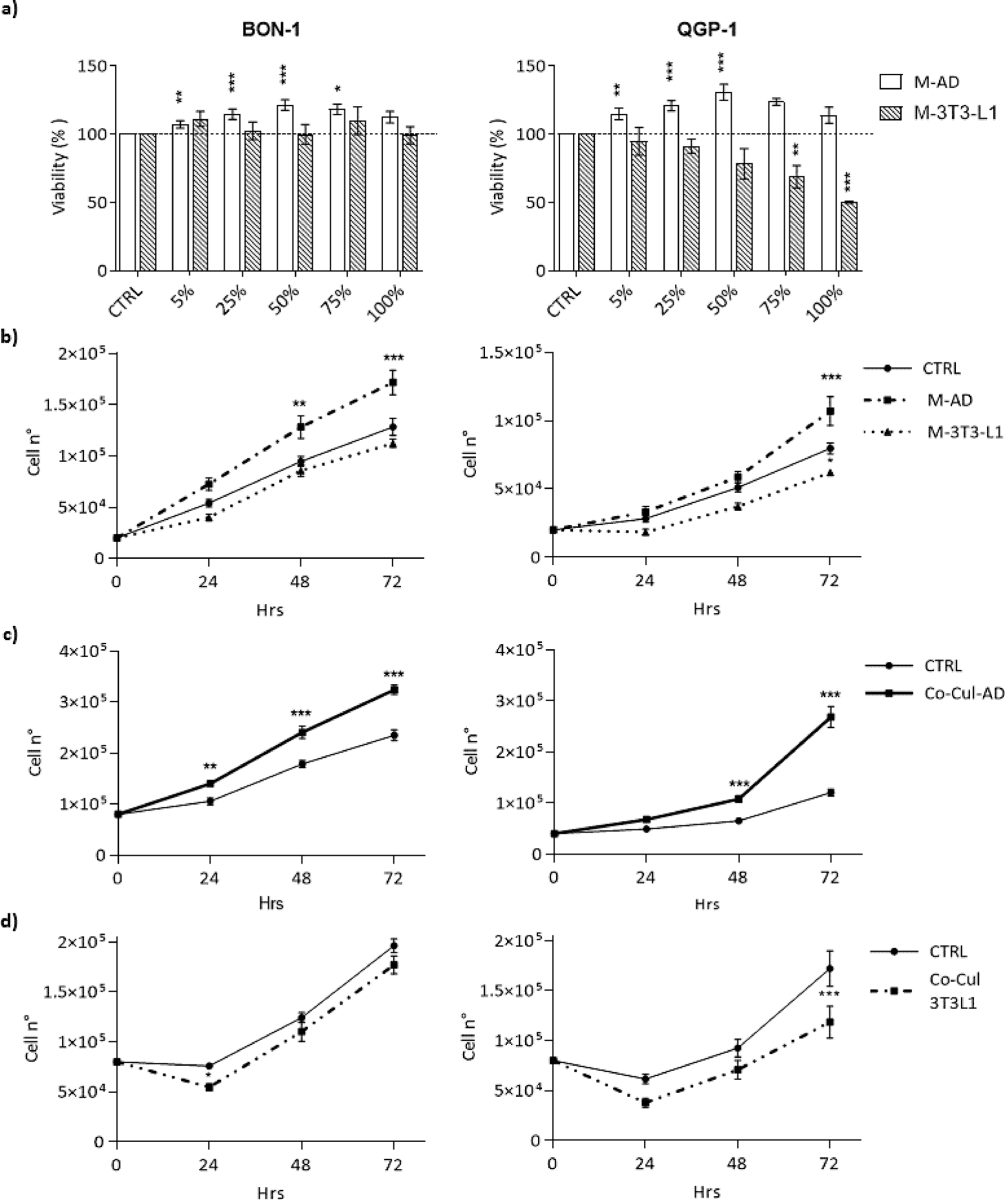
Differential proliferative responses of BON-1 and QGP-1 cells to adipocyte- and fibroblast-derived secretomes, in conditioned medium and co-culture systems. Each panel compares the response of BON-1 cells and QGP-1 cells to distinct experimental conditions involving either adipocytes or fibroblasts. (**a**) MTT assay performed on BON-1 and QGP-1 cells treated for 72 hours with various concentrations (100%, 75%, 50%, 25%, 5%) of conditioned-media from adipocytes or fibroblasts (M-AD or M-3T3–L1), diluted with fresh culture medium. Statistical analysis was performed using one-way ANOVA followed by Dunnett’s post hoc test. (**b**) Proliferation assay based on direct cell counting of BON-1 and QGP-1 cells treated with 50% M-AD or M-3T3–L1 for 24, 48, and 72 hours. Statistical analysis was performed using two-way ANOVA followed by Sidak’s multiple comparisons test. (**c**) Proliferation assay of BON-1 and QGP-1 cells co-cultured with mature adipocytes using a transwell system for 24, 48, and 72 hours. Statistical analysis: two-way ANOVA with Sidak’s post hoc test. (**d**) Proliferation assay of BON-1 and QGP-1 cells co-cultured with undifferentiated fibroblasts (3T3–L1) maintained under fibroblast culture conditions. Statistical analysis: two-way ANOVA with Sidak’s post hoc test. Data are presented as mean ± SEM from at least three independent experiments. *p < 0.05, **p < 0.01, ***p < 0.001 vs. CTRL (cells growth in normal medium)

After investigating the effects of tumor-adipocyte interactions on adipocytes, we next focused on the tumor cell response. To elucidate the potential impact of the adipocytes or fibroblasts secretome on PanNET models, we performed MTT assays by exposing BON-1 and QGP-1 cells to decreasing dilutions of M-AD or M-3T3–L1, each mixed with fresh culture medium (Fig. 3a). Specifically, both BON-1 and QGP-1 cells exhibited a bell-shaped increase in viability in response to M-AD, with a peak enhancement of approximately 30% observed at the 50% dilution (Fig. 3a). M-3T3–L1 did not elicit any significant effect on BON-1 cells (Fig. 3a). On the other hand, QGP-1 cells showed a marked reduction in viability when exposed to undiluted M-3T3–L1 (Fig. 3a). Based on these findings, the 50% M-AD or M-3T3–L1 dilution was selected for subsequent experiments.

To determine whether the observed increase in cell viability was due to enhanced PanNET cellular proliferation, we performed direct cell counting of tumor cells at 24, 48, and 72 hours following incubation with M-AD or M-3T3–L1 in a ratio 1:1 with fresh culture medium (Fig. 3b). Indeed, MTT assays may not always directly correspond to cell number, particularly in the presence of adipocyte-derived factors that can influence mitochondrial metabolism. However, consistent with MTT results, both BON-1 and QGP-1 cell lines showed a statistically significant increase in cell number under M-AD (Fig. 3b). In contrast, exposure to M-3T3–L1 resulted in either no change or a slight reduction in cell counts for BON-1 or QGP-1 cells, respectively (Fig. 3b). We then evaluated whether this growth promoting effect was also maintained under indirect co-culture conditions with adipocytes (Fig. 3c). In this context, a difference in proliferation between the control and co-culture conditions became apparent as early as 24 hours in BON-1 cells, whereas in the M-AD treatment, this effect required 48 hours. Similarly, for QGP-1 cells, a significant increase in proliferation was observed at 48 hours in the co-culture condition, compared to 72 hours needed with M-AD (Fig. 3c). These findings suggest that co-culture is more effective than M-AD alone in inducing growth changes in the studied tumor cell models. In contrast, co-culture with fibroblasts did not significantly affect BON-1 cell proliferation, except at the 24-hour time point (Fig. 3d, left), while a consistent and significant reduction in QGP-1 cell proliferation was observed after 72 hours (Fig. 3d, right).

### Adipocytes do not stimulate the proangiogenic potential of PanNET cells in vivo

To assess the potential impact of adipocytes on PanNET-induced angiogenesis, BON-1 and QGP-1 cells previously cultured either alone or in co-culture with adipocytes were implanted into 2 dpf zebrafish embryos. The *Tg(fli1a:EGFP)*^*y1*^ transgenic line, in which embryos express enhanced green fluorescent protein (EGFP) under the control of the endothelial *fli1a* promoter, was used to visualize the development of the tumor-associated vasculature in vivo [26]. At 1 dpi, a significant pro-angiogenic response, characterized by the formation of new endothelial sprouts extending from the sub-intestinal vessel (SIV) plexus and the common cardinal vein (CCV) toward the tumor implantation site, was observed in embryos engrafted with both PanNET cells compared to control embryos (Suppl. 1). However, the extent of tumor-induced angiogenesis was comparable between embryos implanted with PanNET cells cultured alone and those co-cultured with adipocytes, indicating that the pre-implantation co-culture with adipocytes did not alter the intrinsic pro-angiogenic potential of either BON-1 or QGP-1 cells in our zebrafish xenotransplantation model.

### Reciprocal crosstalk with adipocytes enhances IL-1β secretion in PanNET cells through CXCL12

**Fig. 4:**
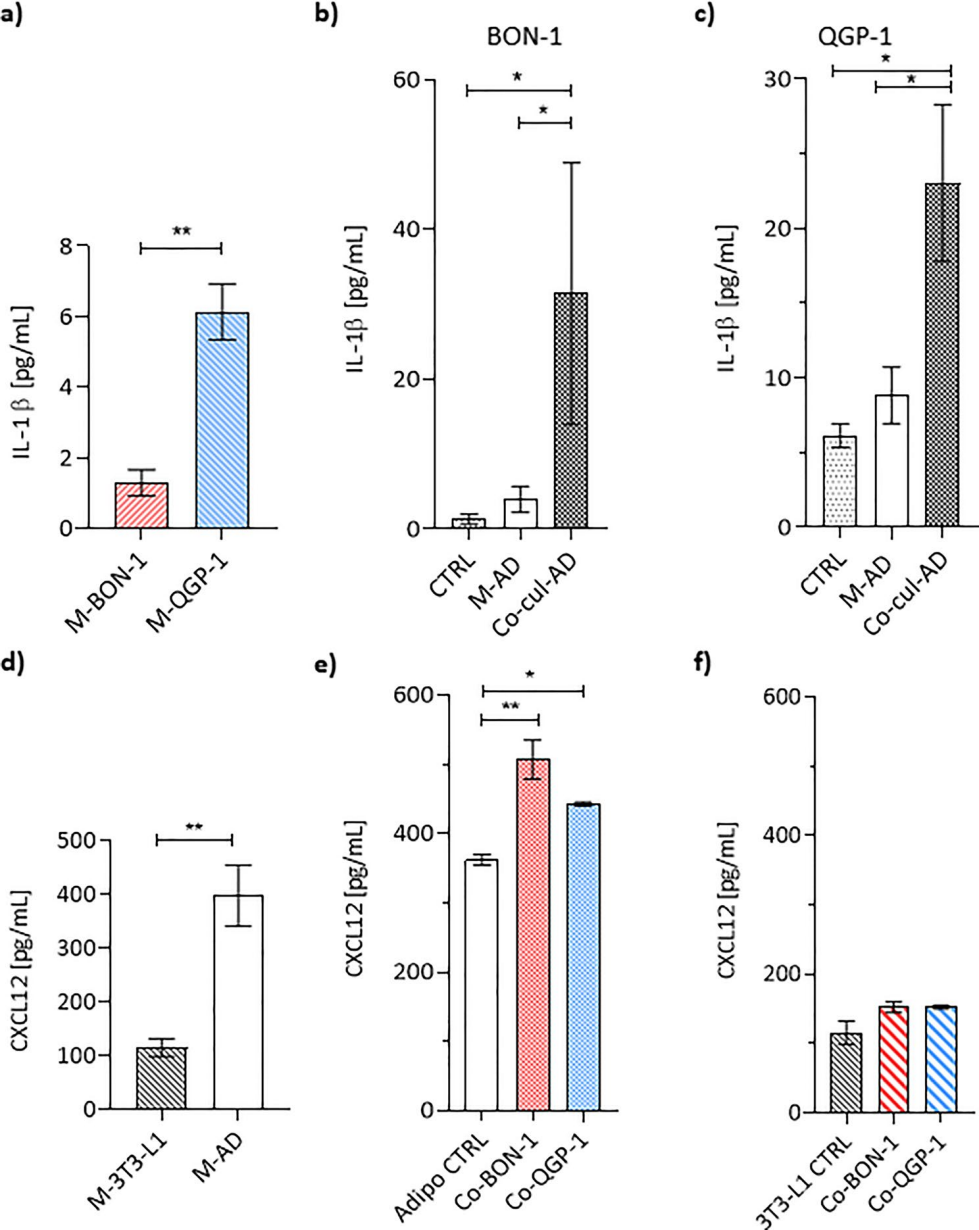
Differential secretion of IL-1β and CXCL12 across conditioned media and co-culture systems. The figure includes multiple panels showing the concentrations (pg/mL) of IL-1β and CXCL12 under various experimental conditions. (**a**) IL-1β levels in conditioned media from M-BON-1 and M-QGP-1 cultures. (**b–c**) IL-1β concentration in supernatants from BON-1 and QGP-1 cells exposed to mature adipocyte-conditioned media (M-AD) or co-cultured with adipocytes, respectively. (**d**) CXCL12 concentration in M-AD compared to M-3T3–L1. e–f) CXCL12 levels in the medium of adipocytes co-cultured with BON-1 or QGP-1 cells (**e**), and fibroblasts co-cultured with the same tumor cell lines (**f**). Statistical analysis was performed using an unpaired *t*-test for comparisons between two groups. Ordinary one-way ANOVA followed by Dunnett’s post hoc test for all other comparisons. Data are presented as mean ± SEM from at least three independent experiments. *p < 0.05, **p < 0.01, **p < 0.001 were considered statistically significant

The acquisition of a fibroblast-like phenotype by adipocytes with pro-inflammatory features may be driven by IL-1β secreted by PanNET cells, as previously described in PDAC [27]. To test this hypothesis, we measured IL-1β levels in the conditioned media collected under various experimental conditions (Fig. 4a–c). IL-1β concentration was significantly higher in M-QGP-1 compared to M-BON-1 (Fig. 4a). A slight but non-significant increase in IL-1β production was also detected when tumor cells were incubated with M-AD (Fig. 4b and c). Under co-culture conditions with adipocytes, IL-1β secretion increased approximately six-fold in BON-1 cells and four-fold in QGP-1 cells compared to controls (Fig. 4b and c).

At the same time, given that CXCL12 is known to act as an upstream regulator of IL-1β expression [28] and may also contribute to tumor growth [29], we investigated the levels of this chemokine in M-AD compared with M-3T3–L1. If adipocyte-induced tumor proliferation is driven by CXCL12, its concentration would be expected to be higher in M-AD than in M-3T3–L1. As shown in Fig. 4d, CXCL12 concentration was approximately four times higher in M-AD compared to M-3T3–L1, supporting the hypothesis that CXCL12 might be a critical mediator of the proliferative effect of the adipocyte secretome (Fig. 4d). Consistently, CXCL12 levels were also markedly elevated under adipocyte co-culture conditions for both BON-1 and QGP-1 cells compared with controls (Fig. 4e). In contrast, CXCL12 expression did not significantly change when tumor cells were co-cultured with fibroblasts (Fig. 4f).

**Fig. 5:**
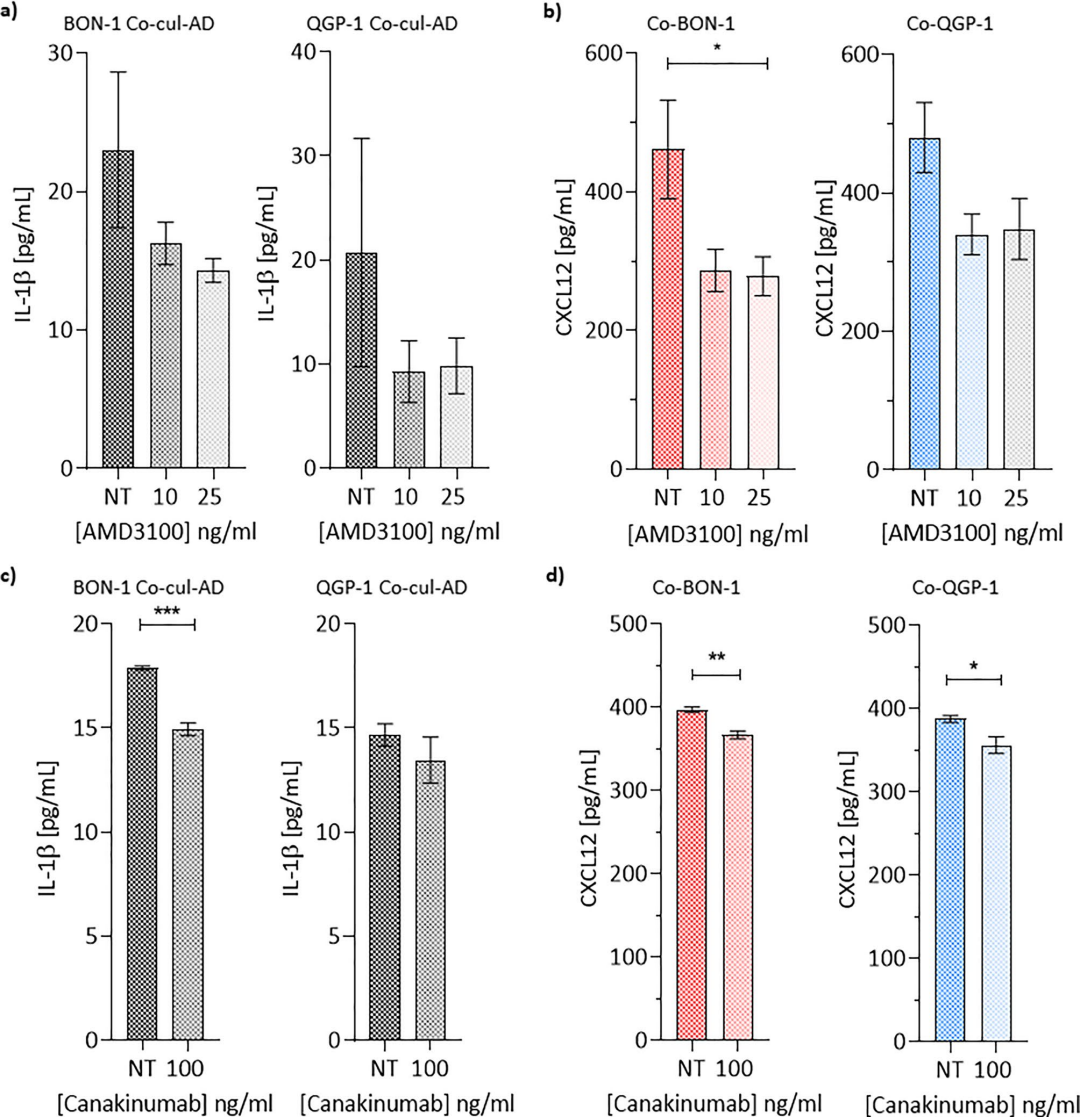
Differential secretion of IL-1β and CXCL12 across conditioned media and co-culture systems after the treatment with AMD3100 and canakinumab. The figure includes multiple panels showing the effects of AMD3100 and canakinumab on the concentrations (pg/mL) of IL-1β and CXCL12 under various experimental conditions **a-b)** IL-1β and CXCL12 levels in co-culture supernatants after treatment with 10 and 25 ng/mL of AMD3100. **c–d)** IL-1β and CXCL12 concentrations in co-cultures treated with 100 ng/mL of canakinumab. Statistical analysis was performed using an unpaired *t*-test for comparisons between two groups. Ordinary one-way ANOVA followed by Dunnett’s post hoc test for all other comparisons. Data are presented as mean ± SEM from at least three independent experiments. *p < 0.05, **p < 0.01, **p < 0.001 were considered statistically significant

To investigate whether IL-1β and CXCL12 production are mutually reinforced through a positive bidirectional crosstalk between adipocytes and tumor cells, we employed two pharmacological inhibitors targeting these distinct components: AMD3100, a selective CXCR4 antagonist that blocks CXCL12-mediated signaling and canakinumab, a monoclonal antibody that directly neutralizes IL-1β [30, 31]. Concentrations of 10 ng/mL and 25 ng/mL for AMD3100, and 100 ng/mL for canakinumab, were selected for the experiments, as they did not significantly affect the viability of either PanNET cell line (Supplementary Fig. 2). As shown in Fig. 5a and b, treatment with AMD3100 induced a trend toward reduced concentrations of both IL-1β and CXCL12 compared to the untreated co-culture condition (NT). A similar trend was observed following treatment with 100 ng/mL canakinumab (Fig. 5c and d). In this case, although IL-1β levels in the QGP-1 co-culture were not significantly altered, a notable 20% reduction in IL-1β concentration was observed in the BON-1 co-culture following treatment. Additionally, CXCL12 levels were reduced by approximately 10% in both co-culture models. These findings further support the notion that CXCL12 and IL-1β production may be functionally interconnected.

### Pharmacological inhibition of the IL-1β–CXCL12 signalling reverts adipocyte dedifferentiation in Co-culture with tumor cells

**Fig. 6:**
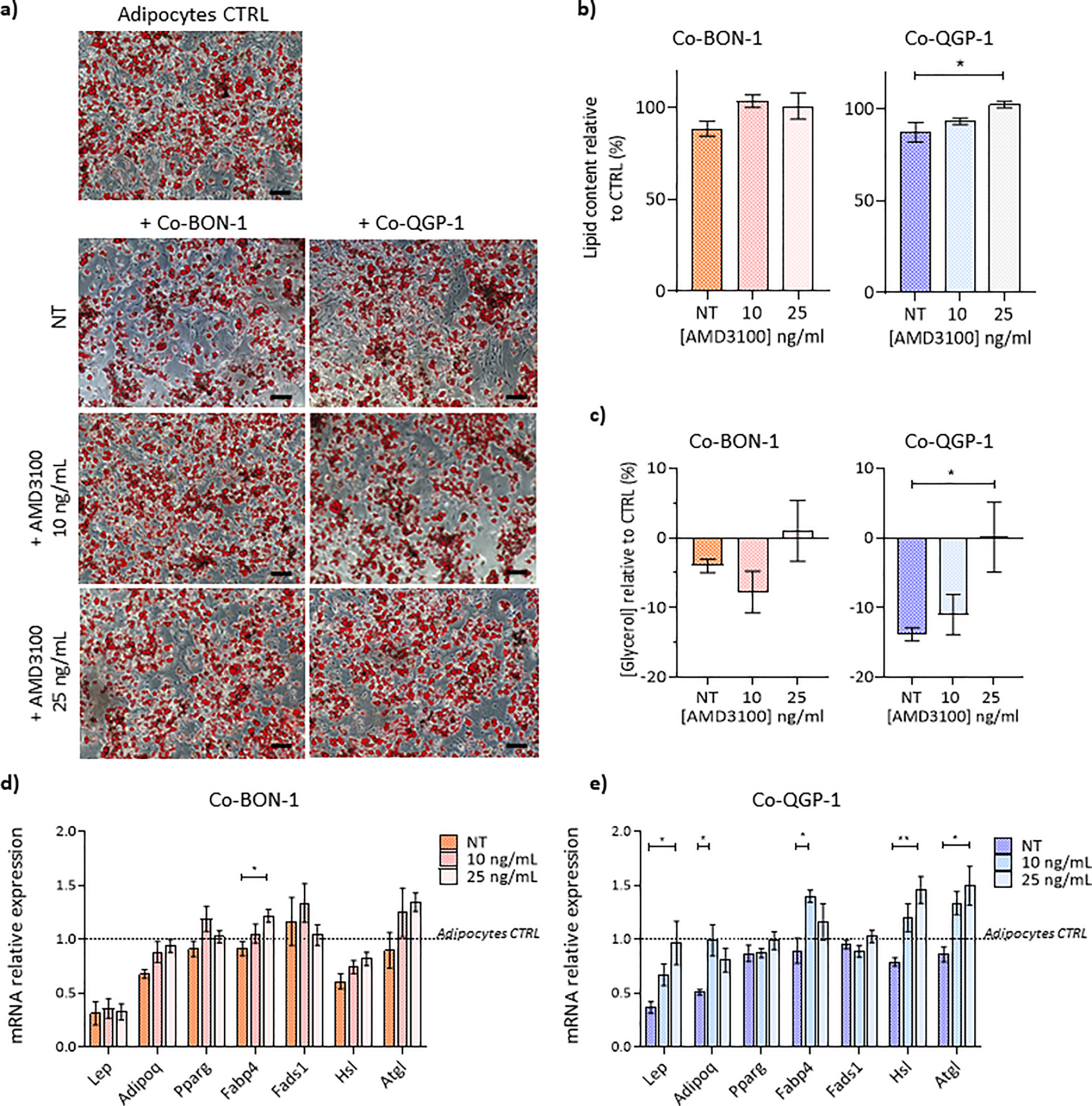
Pharmacological inhibition of CXCL12 with AMD3100 partially restores the adipocyte phenotype in co-culture with BON-1 and QGP-1 tumor cells. (**a**) Representative brightfield images (10× magnification) of differentiated 3T3–L1 adipocytes cultured alone (CTRL), or indirectly co-cultured for 72 hours with PanNET tumor cell lines (co-BON-1 and Co-QGP-1), with or without AMD3100 treatment at 10 ng/mL or 25 ng/mL. Scale bars: 100 μm. (**b**) Quantification of Oil Red O extracted from stained adipocytes is displayed as histograms. (**c**) Lipolytic activity measured by stimulating adipocytes with 10 μM isoproterenol for 2 hours, followed by quantification of glycerol released into the culture medium. (**d-e**) Relative mRNA expression of adipocyte-specific genes (*Lep*, *Adipoq*, *Pparg*, *Fabp4*, *Fads1*, *Hsl*, and *Atgl*) in co-cultured adipocytes with or without AMD3100. Gene expression levels were normalized to a housekeeping gene for BON-1 (**d**) and QGP-1 (**e**) co-cultures. For the analyses shown in panels, all values were first normalized to adipocytes cultured alone. After, treated co-culture samples were compared to untreated co-cultured adipocytes. Statistical analysis was performed using one-way ANOVA followed by Dunnett’s multiple comparisons test. All data are presented as mean ± SEM from at least three independent experiments

Assuming a causative role of IL-1β in driving adipocyte dedifferentiation into CAAs, and considering CXCL12 as a potential upstream inducer of IL-1β, we aimed to disrupt this feedback-like signaling mechanism. We first tested AMD3100 (10 ng/mL and 25 ng/mL) and observed a modest attenuation of the adipocyte dedifferentiation phenotype under co-culture conditions (Fig. 6a). To assess whether treated adipocytes returned to a more physiological state, we first normalized the experimental data to CTRL adipocytes that had never been exposed to tumor cells. We then compared the treated samples to untreated co-cultured adipocytes (NT). Adipocytes treated with the CXCR4 antagonist and co-cultured with BON-1 cells displayed a slight tendency to regain their physiological phenotype. In contrast, with QGP-1 cells, treated adipocytes displayed a complete restoration of lipid content compared to NT, as evidenced by Oil Red O staining and optical microscopy, and showed a significant increase in glycerol release respect to NT to levels more similar to those of untreated control adipocytes, especially at the higher drug concentration (Fig. 6a, b and c). As for the expression of key adipocyte markers, a general dose-dependent trend was observed, with all targets showing a tendency to increase their expression in treated conditions compared to NT. However, in the BON-1 co-culture condition, only *Fabp4* expression showed a significant increase following treatment with AMD3100, reaching levels that were both significantly higher than untreated co-cultured adipocytes and closer to those of the control group (Fig. 6d). In contrast, in the QGP-1 co-culture model, multiple markers, *Lep, Adipoq, Fabp4, Hsl*, and *Atgl,* showed significant upregulation following treatment, with expression levels trending toward adipocytes CTRL (fold change 1), and significantly differing from NT co-cultured adipocytes (Fig. 6e). This partial reversion may be attributed to the indirect downregulation of IL-1β following CXCL12 inhibition.

**Fig. 7:**
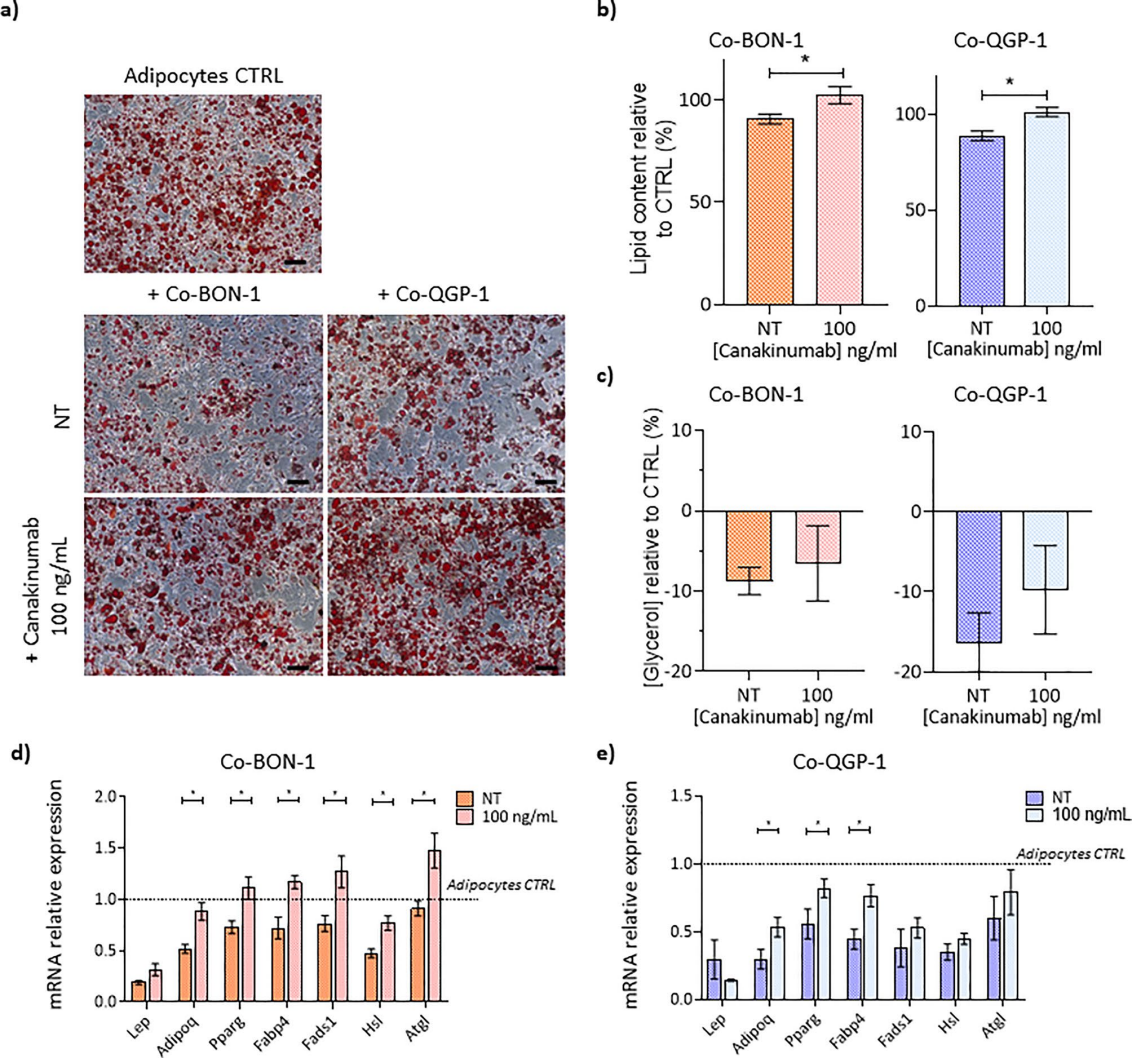
Targeting IL-1β with canakinumab prevents adipocyte dedifferentiation induced by co-culture with BON-1 and QGP-1 cells. (**a**) Representative brightfield images (10× magnification) of differentiated 3T3–L1 adipocytes maintained in monoculture (CTRL) or indirectly co-cultured with PanNET tumor cells (co-BON-1 and Co-QGP-1) for 72 hours, with or without treatment using 100 ng/mL canakinumab. Scale bars: 100 μm. (**b**) Quantification of Oil Red O extracted from stained adipocytes is displayed as histograms. (**c**) Assessment of lipolytic response following 2-hour stimulation with 10 μM isoproterenol, measured by quantification of glycerol released into the culture medium. (**d-e**) Analysis of adipocyte gene expression profiles by RT-qPCR. Transcripts for *Lep*, *Adipoq*, *Pparg*, *Fabp4*, *Fads1*, *Hsl*, and *Atgl* were measured and normalized to a reference gene for BON-1 (**d**) and QGP-1 (**e**) cells. Experimental values were first normalized to the baseline levels observed in adipocytes cultured alone. Treated co-culture samples were then statistically compared to untreated co-cultures. Statistical significance was assessed using one-way ANOVA followed by Dunnett’s post-hoc test. Data represent the mean ± SEM from a minimum of three independent experiments. *p < 0.05, **p < 0.01, **p < 0.001 were considered statistically significant

We next evaluated the effects of canakinumab. Canakinumab significantly restored intracellular lipid content in co-cultured BON-1 and QGP-1 adipocytes, although glycerol release into the medium was not significantly altered (Fig. 7a, b and c). Importantly, canakinumab treatment led to a marked recovery in the expression of multiple adipocyte-specific gene markers (Fig. 7d and e). These effects were particularly evident in BON-1 co-cultures, indicating that a 100 ng/mL dose of canakinumab was sufficient to counteract IL-1β-driven dedifferentiation in this model (Fig. 7d). In contrast, in QGP-1 co-cultures, the same treatment significantly upregulated only *Adipoq*, *Pparg*, and *Fabp4,* genes strongly associated with adipocyte identity (Fig. 7e).

### Targeting CXCL12 reduces tumor proliferation

**Fig. 8:**
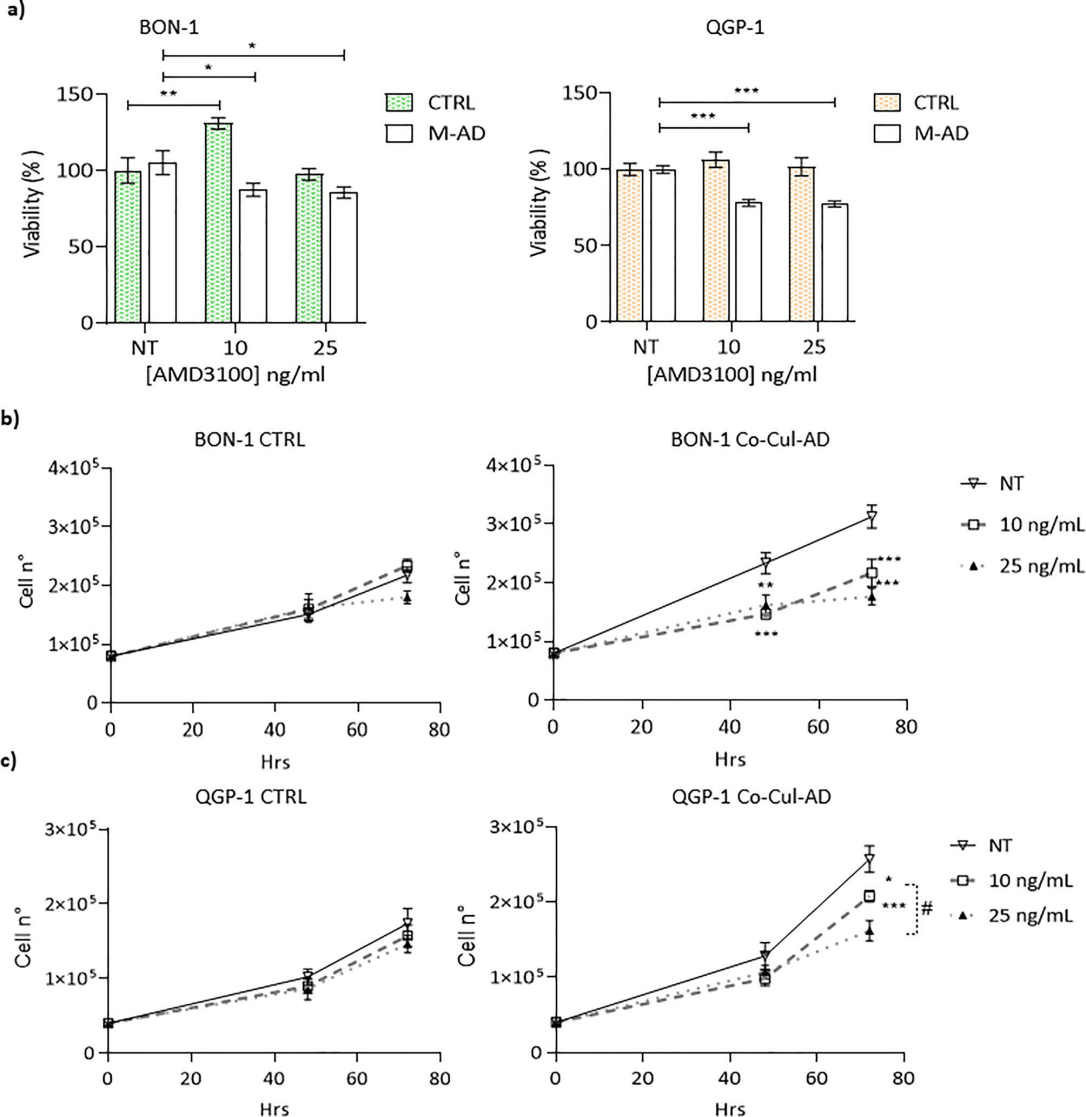
Pharmacological treatment with AMD3100 inhibits the proliferative response of BON-1 and QGP-1 cells in adipocyte co-culture systems. (**a**) MTT assay performed on BON-1 and QGP-1 cells treated for 62 hours with 10 or 25 ng/mL AMD3100 diluted in conditioned medium from adipocytes (M-AD) or standard culture medium (CTRL). **b–c**) Proliferation assays on BON-1 (**b**) and QGP-1 (**c**) cells cultured in standard growth medium (CTRL) or indirectly co-cultured with adipocytes (co-cul-AD) using a transwell system for 72 hours, in the presence or absence of 10 or 25 ng/mL AMD3100. Data in panel a were analyzed using one-way ANOVA followed by Dunnett’s post hoc test. Data in panels b, and c were analyzed using two-way ANOVA followed by Sidak’s post hoc test. Results are presented as mean ± SEM from at least three independent experiments. *p < 0.05, **p < 0.01, *** *p* < 0.001 vs. NT (cells grown in standard medium with vehicle control); # *p* < 0.05 10 ng/ml vs 25 ng/ml

We next focused on evaluating the effects of disrupting the IL-1β/CXCL12 axis on PanNET proliferation. In the MTT assay, AMD3100 did not display any cytotoxic effects in either BON-1 and QGP-1 cells under standard growth conditions (CTRL; Fig. 8a). Rather, treatment with 10 ng/mL AMD3100 significantly increased cell viability in BON-1. However, treatment with AMD3100 at 10 ng/mL and 25 ng/mL significantly reduced the viability in BON-1 and QGP-1 cells cultured with M-AD (Fig. 8a). This supports the hypothesis that AMD3100 interferes specifically with CXCL12 signaling derived from the adipocyte secretome, rather than impairing baseline tumor cell viability. In standard culture conditions (CTRL), treatment with AMD3100 did not significantly affect the proliferation of either BON-1 or QGP-1 cells (Fig. 8b and c). In contrast, under co-culture conditions with adipocytes, AMD3100 markedly reduced the proliferation of both cell lines in a dose-dependent manner (Fig. 8b and c). Specifically, both BON-1 and QGP-1 cells exhibited a clear dose-dependent response to the drug, with the highest concentration leading to an approximately 40% reduction in tumor proliferation under co-culture conditions in both cases.

**Fig. 9:**
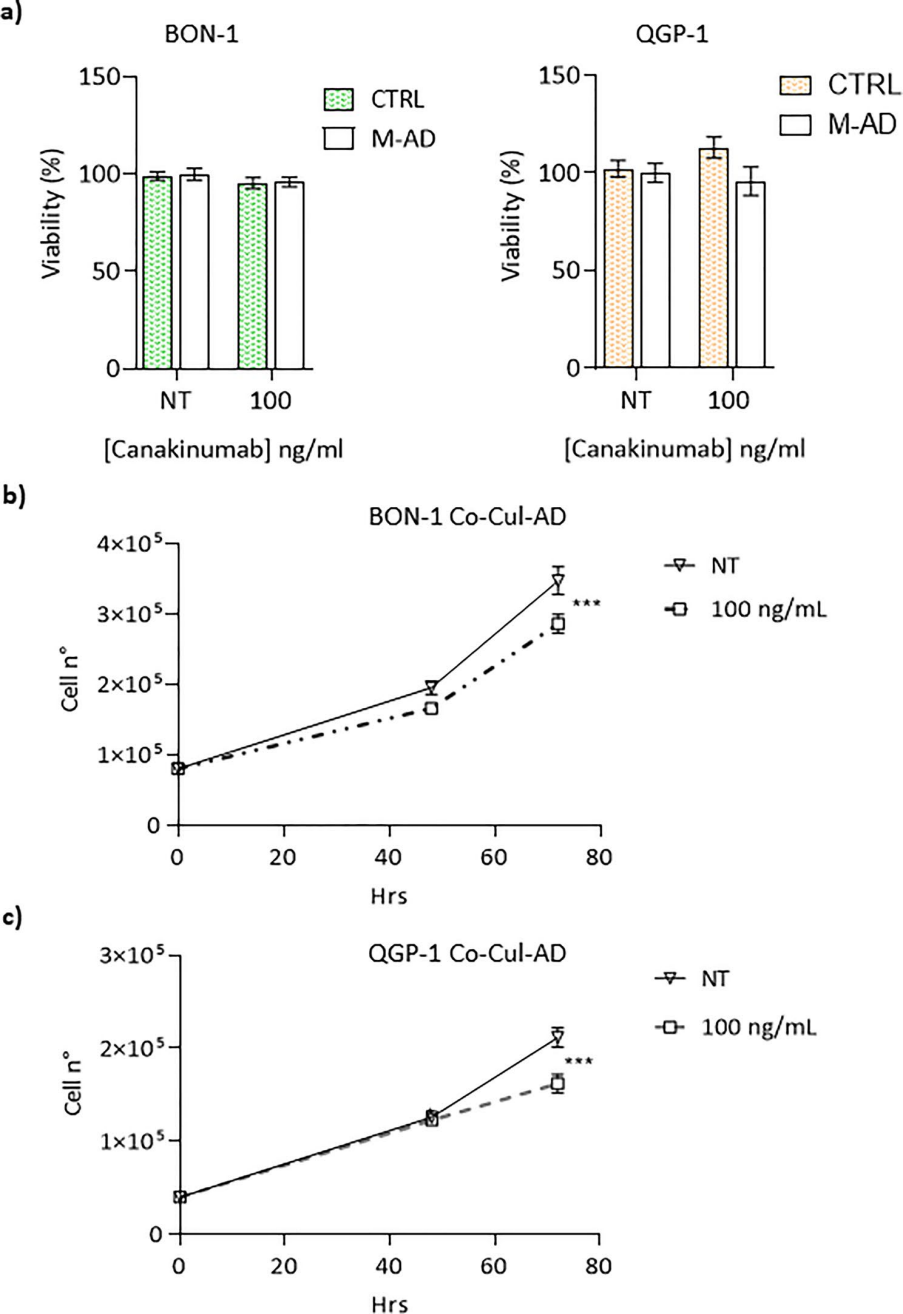
Pharmacological treatment with canakinumab inhibits the proliferative response of BON-1 and QGP-1 cells in adipocyte co-culture systems. (**a**) MTT assay performed on BON-1 and QGP-1 cells treated for 72 hours with 100 ng/mL canakinumab diluted in M-AD or standard medium. (**b**–**c**) Proliferation assays on BON-1 (**b**) and QGP-1 (**c**) Cells cultured in standard medium or indirectly co-cultured with adipocytes in the presence or absence of 100 ng/mL canakinumab. Data in panel a were analyzed using one-way ANOVA followed by Dunnett’s post hoc test. Data in panels b and c were analyzed using two-way ANOVA followed by Sidak’s post hoc test. Results are presented as mean ± SEM from at least three independent experiments. *** *p* < 0.001 vs. NT (cells grown in standard medium with vehicle control)

We then tested the effect of canakinumab, based on the rationale that IL-1β inhibition would indirectly reduce CXCL12 levels (Fig. 5b) and consequently decrease tumor proliferation. In monoculture incubated with M-AD, canakinumab had again no effect on the proliferation of either BON-1 or QGP-1 cells, consistent with the absence of direct CXCL12 blockade (Fig. 9a). However, in co-culture with adipocytes, canakinumab significantly reduced tumor proliferation by approximately 16% in BON-1 cells and 25% in QGP-1 cells, likely through inhibition of IL-1β–dependent CXCL12 production (Fig. 9b and c).

## Discussion

In several tumors adipocytes have recently emerged as metabolically active endocrine cells within the tumor microenvironment, capable of influencing tumor initiation, progression, and metastasis [16, 17, 32]. Our study extends this concept to PanNETs, providing evidence that adipocyte–tumor interactions represent a previously underappreciated, yet biologically relevant, component of PanNET biology.

Here, we demonstrate that PanNET cells actively induce the trans-differentiation of mature adipocytes into CAAs. This process is characterized by a marked loss of the adipocyte differentiation program, as shown by the downregulation of canonical adipogenic markers (*Lep, Adipoq, Pparg*, and *Fabp4*) and genes involved in lipogenesis and triglyceride metabolism (*Fads1, Hsl* and *Atgl*), together with a significant reduction in lipid droplet content. Morphologically, these changes are accompanied by the acquisition of a fibroblast-like phenotype, consistent with previous descriptions of CAAs in other tumors [16, 31]. Notably, adipocyte reprogramming was markedly more pronounced under indirect co-culture conditions than upon exposure to tumor-derived conditioned media alone. Indeed, conditioned media derived from QGP-1 cells (M-QGP-1), but not from BON-1 cells, induced only a partial adipocyte reprogramming toward a CAA-like phenotype. This finding suggests that QGP-1 cells constitutively secrete soluble factors capable of initiating adipocyte phenotypic changes; however, their concentration and/or temporal persistence appear insufficient to fully recapitulate the extensive dedifferentiation observed under co-culture conditions. Moreover, it should be considered that conditioned media were used in combination with standard culture medium (1:1 dilution), which may have reduced the effective concentration of tumor-derived factors, thereby contributing to the attenuated response. In any case, these data underscore the importance of a dynamic and reciprocal paracrine interaction between adipocytes and tumor cells, supporting the notion that sustained bidirectional signaling is required to drive complete adipocyte reprogramming. In this context, the indirect co-culture system represents a biologically relevant in vitro model, mimicking the in vivo scenario in which PanNET cells and adipocytes are anatomically proximal.

To identify the signalling factors potentially triggering the dedifferentiation process, we sought to additionally characterize the phenotypic changes occurring in adipocytes. One possibility was that our adipocytes were undergoing a process known as adipocyte-to-mesenchymal transition (AMT), through which mature adipocytes progressively acquire mesenchymal, fibroblast-like features which are often referred to as fat fibroblasts or dedifferentiated fat cells or adipocyte-derived fibroblasts [33–35]. Adipocyte-derived fibroblasts have been identified in many in vivo pathological conditions, such as breast and pancreatic cancer, associated with tumor progression and are not restricted to in vitro induction [34, 36]. Moreover, it is well established that suppression of PPARγ expression, combined with exposure to tumor-derived inflammatory cytokines, can initiate AMT [36]. Interestingly, in PanNETs, histopathological analyses frequently reveal a dense fibrotic stroma enriched in fibroblast-like cells [37, 38]. In this context, if a fraction of adipocytes transdifferentiated via AMT, the surrounding adipose tissue should be regarded not as a passive metabolic bystander, but rather as an active participant in tumor progression [39, 40]. However, unlike other tumor types in which adipocytes are adjacent to malignant epithelial cells, PanNETs appear to represent a distinct biological context [33]. Although the pancreas is surrounded by abundant peripancreatic adipose tissue, PanNETs typically develop as well-circumscribed lesions and direct contact with adipocytes is generally limited to specific regions, particularly in areas of adipocyte infiltration within the pancreatic parenchyma. Instead, they develop within a chronically pro-inflammatory, highly vascularized, and cytokine-rich microenvironment, which may indirectly influence adipocyte phenotype and function [41, 42]. Given this inflammatory setting, it was plausible that adipocytes exposed to PanNET-derived soluble factors could undergo a gradual phenotypic transition, first acquiring characteristics of CAAs and subsequently adopting an iCAF-like profile, characterized by the secretion of high levels of immunomodulatory cytokines, as observed in PDAC [43–46].

To test this hypothesis, we examined the expression and subcellular localization of F-actin and α-SMA, two canonical markers of myofibroblastic differentiation, as well as IL-6, a hallmark of the iCAF phenotype, in our co-culture model. We observed a marked downregulation of α-SMA expression and its dissociation from F-actin stress fibers, accompanied by a significant increase in IL-6 secretion. Collectively, these changes indicate the acquisition of a pro-inflammatory CAF-like phenotype, also supported by the increased secretion of CXCL12, rather than a contractile or fibrotic stromal program [38]. However, the star-like morphology and the presence of stress fibers do not pertain to the classical iCAF presentation, although supporting the transition from CAA to reactive CAF. In this scenario, tumor-derived cues such as IL-1β would act as the principal modulators of the adipocyte reprogramming process [47]. The observed phenotype is most plausibly interpreted as a transitional state, positioning these cells as intermediates along a continuum between CAAs and iCAFs. This may be due either to the relatively short experimental timeframe of our study, or represent a specific CAF subpopulation induced by the intrinsic characteristics of PanNET microenvironment. Indeed, multi-omic studies recently revealed the presence of several CAFs phenotypes that may dynamically evolve into one another and whose characteristics may partially overlap with other healthy and tumor associated cell types [48].

In PanNET models, Lai et al. identified iCAFs both in vivo and in vitro and demonstrated their crucial role in disease progression, correlating the highest density of activated fibroblasts as a significant predictor of clinical aggressiveness [49]. Mechanistically, PanNET cells secreting IL-1 induced an inflammatory transcriptional program in CAFs, characterized by robust upregulation of CXCL12, which in turn promoted tumor cell proliferation and motility [49]. Although our study focused on adipocytes rather than fibroblasts, a similar paracrine mechanism appears to occur. Consequently, we hypothesized that adipocyte–tumor crosstalk could preferentially sustain tumor cell growth. On the other hand, PanNETs are typically highly vascularized neoplasms, and their growth is known to be strongly dependent on angiogenesis, which represents a well-established hallmark of these tumors and a validated therapeutic target. For this reason, we also investigated whether adipocyte-derived signals could modulate tumor-induced angiogenesis in vivo using the zebrafish model. However, no significant effect was observed. In contrast, adipocyte-derived factors significantly enhanced PanNET cell proliferation, as confirmed by both MTT and direct cell counting assays, with a more pronounced and earlier effect under co-culture conditions.

We found that PanNET cells secrete IL-1β. This process was markedly enhanced under adipocyte co-culture conditions, suggesting that adipocyte-derived signals amplify inflammatory cytokine production by PanNET cells. In parallel, we identified CXCL12 as a critical downstream effector of adipocyte reprogramming and a major contributor to tumor-promoting crosstalk. Adipocytes secreted substantially higher levels of CXCL12 compared with fibroblasts, and CXCL12 production was selectively increased under adipocyte–PanNET co-culture conditions.

CXCL12 plays a pivotal role in promoting tumor proliferation, migration and establishment of an inflammation niche across multiple cancer types [50–52]. In breast and ovarian cancers, stromal cell–derived CXCL12 enhances tumor proliferation, migration, and immune evasion via CXCR4/CXCR7-dependent PI3K/AKT and MAPK/ERK pathways [51, 53, 54]. Similarly, in PDAC, the CXCL12–CXCR7 signaling promotes tumor cell migration and invasion through the mTOR and Rho/ROCK pathways [55]. In neuroendocrine tumors, CXCR4 has been associated with tumor aggressiveness, dedifferentiation, and poor prognosis. Moreover, CXCR4 has recently been proposed as a novel prognostic marker in PanNETs [56–59].

As for IL-1β, elevated levels of this cytokine have been correlated with increased tumor aggressiveness and reduced therapeutic responsiveness in several malignancies and inflammatory stromal remodelling, such as PDAC, prostate cancer, and PanNETs [60, 61]. Genetic evidence further supports the involvement of IL-1β in PanNET pathophysiology: the IL1B−511 T variant has been associated with an increased risk of developing PanNETs, particularly the functional subtypes that actively secrete hormones [42, 62]. However, IL-1β is typically undetectable in the peripheral blood of PanNETs affected patients, suggesting that IL-1β acts locally within the tumor microenvironment [62].

In our model, the functional relevance of the IL-1β/CXCL12 axis was supported by experiments using AMD3100 and canakinumab. Notably, although these treatments only moderately reduced the levels of secreted CXCL12 and IL-1β, this is consistent with their mechanism of action, as both compounds interfere with signaling activity rather than directly suppressing cytokine production. Therefore, their biological relevance is better reflected by the downstream functional effects. In particular, inhibition of CXCR4 signaling with AMD3100 effectively prevented adipocyte dedifferentiation, reduced CXCL12 and IL-1β levels, and significantly attenuated PanNET proliferation under co-culture conditions. Notably, AMD3100 had no appreciable effects on tumor cell proliferation in monoculture, indicating that its antitumor activity is primarily mediated through disruption of microenvironment-derived signals rather than direct cytotoxicity. Similarly, blockade of IL-1β signaling with canakinumab partially reversed adipocyte reprogramming and reduced tumor cell proliferation, although its effects were less pronounced than those achieved by direct CXCL12 inhibition. Together, these data suggest that CXCL12 enhanced PanNET cell proliferation and further stimulated IL-1β release, thereby establishing a positive feedback-like mechanism between tumor-derived IL-1β and adipocyte-derived CXCL12. This reciprocal signaling axis provides a mechanistic framework linking adipocyte dedifferentiation to tumor growth and inflammatory microenvironment remodeling.

From a translational perspective, these findings highlight the IL-1β/CXCL12 signaling axis as a potential therapeutic vulnerability in PanNETs. CXCR4 inhibition appears particularly attractive, as it simultaneously targets tumor cells and their supportive stromal compartment. Notably, AMD3100 is already clinically approved for hematological indications and has shown antitumor activity in several solid malignancies [63], supporting the feasibility of repurposing CXCR4-targeting strategies in PanNETs. Although IL-1β inhibition alone may be insufficient to fully suppress tumor growth, its ability to modulate stromal reprogramming suggests that combination approaches targeting both inflammatory and chemokine-driven pathways may yield synergistic benefits.

Despite the strengths of our study, several limitations should be acknowledged. Our conclusions are based on in vitro models using established cell lines and indirect co-culture systems, which cannot fully recapitulate the cellular complexity and spatial organization of the in vivo pancreatic microenvironment. Adipocyte experiments were performed using 3T3–L1 cells, a well-established murine model of adipogenesis that, does not fully reflect the biological features of human visceral adipose tissue. Therefore, validation in primary human adipocytes or patient-derived models will be essential to strengthen the translational relevance of our results in future studies. Moreover, the potential influence of tumor grade, hormonal activity, and genetic background on adipocyte reprogramming was not addressed and warrants further investigation.

In conclusion, our data uncover a previously underrecognized role of adipose tissue as an active stromal component in PanNET biology. By orchestrating a bidirectional IL-1β/CXCL12 crosstalk, PanNET cells and adipocytes appear to contribute to the establishment of a pro-inflammatory, tumor-supportive microenvironment that promotes tumor proliferation. These findings not only expand current understanding of tumor–stroma interactions in PanNETs but seem to point toward a microenvironment-centered therapeutic strategy deserving further preclinical and clinical exploration.

## Electronic supplementary material

Below is the link to the electronic supplementary material.

Supplementary Material 1

Supplementary Material 2

Supplementary Material 3

## Data Availability

The dataset supporting the conclusions of this article is available in the [Zenodo] repository, https://10.5281/zenodo.18349614.
