# Supplementary figures and images for: IL-1β/CXCL12 signalling orchestrates adipocyte–pancreatic neuroendocrine tumor crosstalk
Source: J Transl Med. 2026 Jun 18;24:807. doi: 10.1186/s12967-026-08428-z (PMC13292532; doi:10.1186/s12967-026-08428-z)

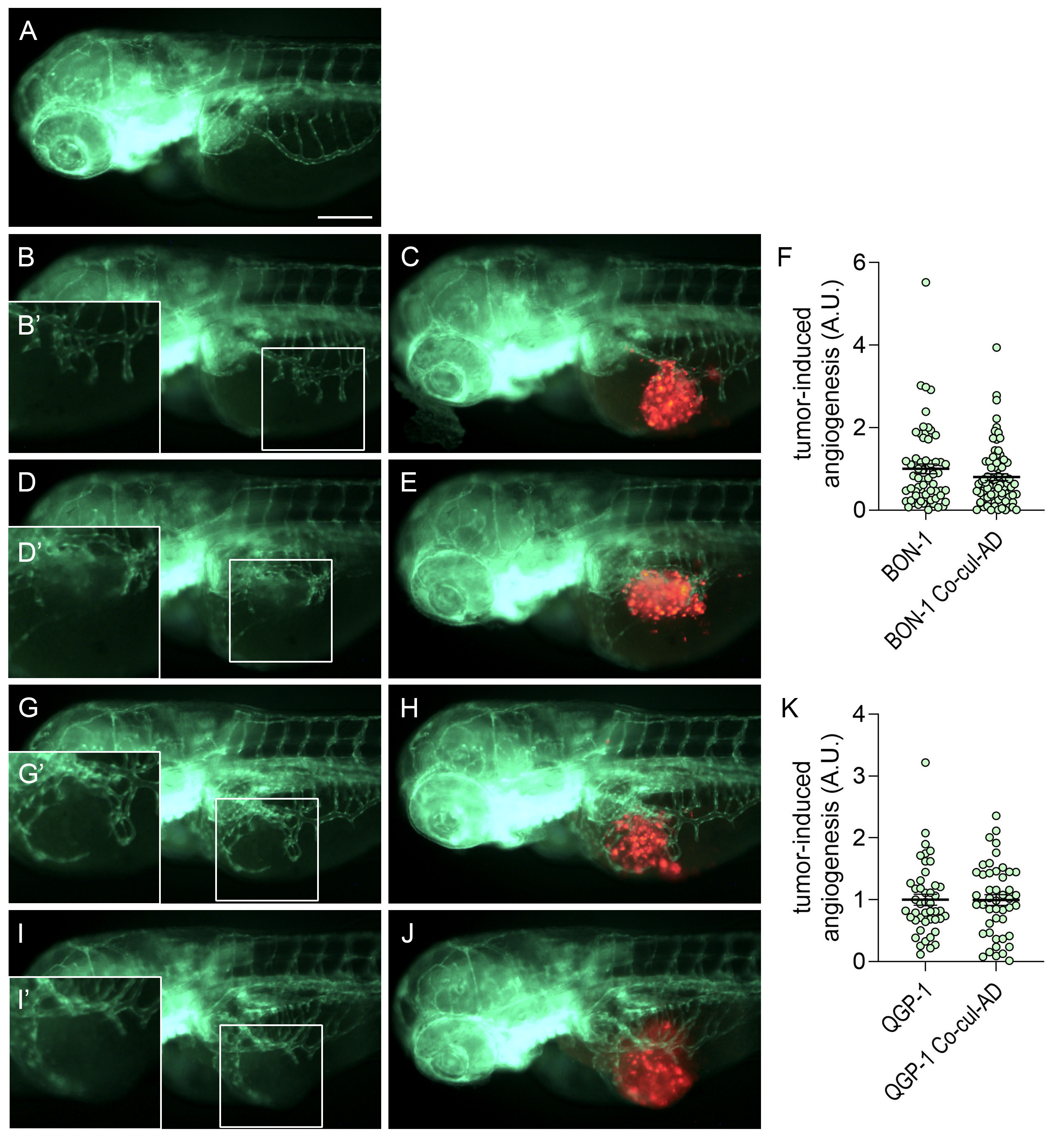

Supplement: Supplementary file 1 — Supplementary Material 1 [file 12967_2026_8428_MOESM1_ESM.jpg]

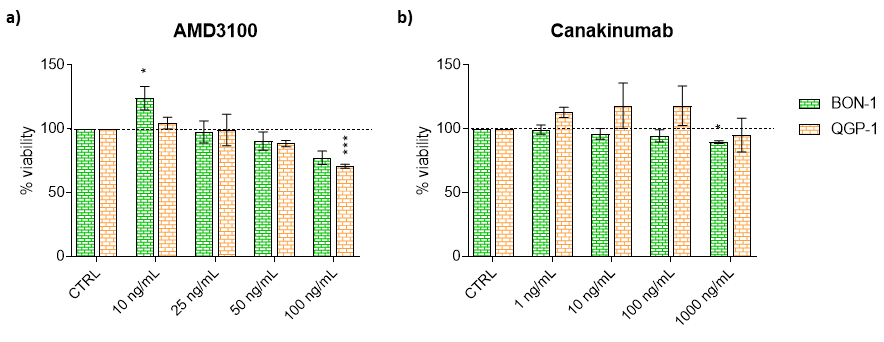

Supplement: Supplementary file 2 — Supplementary Material 2 [file 12967_2026_8428_MOESM2_ESM.png]
