# Supplementary material for: IL-1β/CXCL12 signalling orchestrates adipocyte–pancreatic neuroendocrine tumor crosstalk
Source: J Transl Med. 2026 Jun 18;24:807. doi: 10.1186/s12967-026-08428-z (PMC13292532; doi:10.1186/s12967-026-08428-z)
Supplement: Supplementary file 3 — Supplementary Material 3 [file 12967_2026_8428_MOESM3_ESM.docx]

**Suppl 1. *In vivo* impact of adipocytes on PanNET-induced angiogenesis.** Panels A–E and G–J show representative epifluorescence images of *Tg(fli1a:EGFP)^y1^* embryos at 1 day post-implantation (dpi). Embryos were injected with PBS only, corresponding to the cell suspension vehicle (control; A), or implanted with red-labeled BON-1 cells cultured alone (B, B’, C) or co-cultured with adipocytes (BON-1 Co-cul-AD; D, D’, E). Similarly, red-labeled QGP-1 cells were implanted after being cultured alone (G, G’, H) or in co-culture with adipocytes (QGP-1 Co-cul-AD; I, I’, J). To enhance visualization of tumor-induced angiogenesis (green), the red fluorescence channel was omitted in panels B, B’, D, D’, G, G’, I, and I’. Panels B’, D’, G’, and I’ show digital magnifications of the regions delineated by white boxes.

Quantification of tumor-induced angiogenesis, shown in graphs F and K, revealed that all implanted cell lines similarly promoted endothelial sprouting from the sub-intestinal vessel (SIV) plexus and the common cardinal vein (CCV). Co-culture with adipocytes did not enhance the pro-angiogenic capacity of either BON-1 or QGP-1 cells. Data were normalized to the mean angiogenic response observed in embryos implanted with BON-1 and QGP-1 cells cultured alone, which was arbitrarily set to 1.0. All images are oriented with the rostral end to the left and the dorsal side at the top. Scale bar in panel A = 100 μm. Data in graphs F and K are presented as mean ± standard error of the mean (SEM). Abbreviation: A.U., arbitrary units.

**Suppl. 2:** Cell viability of BON-1 (green) and QGP-1 (yellow) cell lines following treatment with AMD3 100 (a) and canakinumab (b).
